# Dynamic sub-route-based self-adaptive beam search Q-learning algorithm for traveling salesman problem

**DOI:** 10.1371/journal.pone.0283207

**Published:** 2023-03-21

**Authors:** Jin Zhang, Qing Liu, XiaoHang Han

**Affiliations:** 1 School of Computer and Information Engineering, Henan University, Kaifeng, Henan, China; 2 Henan Key Laboratory of Big Data Analysis and Processing, Henan University, Kaifeng, Henan, China; Chang Gung University, TAIWAN

## Abstract

In this paper, a dynamic sub-route-based self-adaptive beam search Q-learning (DSRABSQL) algorithm is proposed that provides a reinforcement learning (RL) framework combined with local search to solve the traveling salesman problem (TSP). DSRABSQL builds upon the Q-learning (QL) algorithm. Considering its problems of slow convergence and low accuracy, four strategies within the QL framework are designed first: the weighting function-based reward matrix, the power function-based initial Q-table, a self-adaptive *ε-beam* search strategy, and a new Q-value update formula. Then, a self-adaptive beam search Q-learning (ABSQL) algorithm is designed. To solve the problem that the sub-route is not fully optimized in the ABSQL algorithm, a dynamic sub-route optimization strategy is introduced outside the QL framework, and then the DSRABSQL algorithm is designed. Experiments are conducted to compare QL, ABSQL, DSRABSQL, our previously proposed variable neighborhood discrete whale optimization algorithm, and two advanced reinforcement learning algorithms. The experimental results show that DSRABSQL significantly outperforms the other algorithms. In addition, two groups of algorithms are designed based on the QL and DSRABSQL algorithms to test the effectiveness of the five strategies. From the experimental results, it can be found that the dynamic sub-route optimization strategy and self-adaptive *ε-beam* search strategy contribute the most for small-, medium-, and large-scale instances. At the same time, collaboration exists between the four strategies within the QL framework, which increases with the expansion of the instance scale.

## 1. Introduction

For a set of given cities, the traveling salesman problem (TSP) is finding the shortest route along which a salesman visits all of the cities exactly once before returning to the starting point. The TSP is a well-known combinatorial optimization problem with applications in many fields [1], such as transportation, circuit board design, production scheduling, and logistics distribution.

As a traditional NP-hard problem, numerous approaches have been proposed to solve the TSP, most of which use exact and heuristic algorithms. Exact algorithms include branch-and-bound (BnB), cut-plane, integer programming, and dynamic programming, all of which are used to obtain the global optimal solution by continuous iteration. For example, Pekny et al. (1990) [2] and Pesant et al. (1998) [3] proposed the BnB method and its variants for the TSP problem, and the famous TSP solver Corconde (http://www.math.uwaterloo.ca/tsp/concorde/index.html) is based on the BnB algorithm. Sanches et al. (2017) [4] proposed a partitioned cross-improvement initial solution method to speed up Corconde. However, since the time cost of the exact algorithm increases exponentially with the size of the instance, they are not suitable for large-scale applications.

Heuristic algorithms are widely used because of their high computational efficiency, and they can obtain a sub-optimal solution in a reasonable timeframe. Representative heuristic algorithms include the Lin-Kernighan heuristic (LKH), ant colony optimization (ACO) algorithm, genetic algorithm (GA), particle swarm optimization (PSO) algorithm, whale optimization algorithm (WOA), and gray wolf optimization (GWO). Based on this, various improved algorithms have been studied for solving the TSP problem. For example, by modifying the heuristic rules of LKH to improve its search strategy, Helsgaun (2000) [5] proposed an improved LKH algorithm. Ebadinezhad et al. (2020) [6] proposed an ACO algorithm that included a dynamic evaporation strategy (DEACO). Wang et al. (2022) [7] proposed a fine-grained fast parallel GA algorithm based on a ternary optical computer, and Zheng et al. (2022) [8] proposed a transfer learning-based PSO algorithm. Zhang et al. (2020) [9] proposed a variable neighborhood discrete WOA algorithm (VDWOA), while Panwar et al. (2021) [10] proposed a novel discrete GWO algorithm. However, heuristic algorithms are mostly based on random search, which lacks both an ability to learning and a theoretical foundation. They also usually require unique heuristic rules for a given problem. For this reason, designing unified solutions for combinatorial optimization problems such as TSP has become a popular research topic in machine learning.

As one of the main machine-learning methods, reinforcement learning (RL) has strong decision making and autonomous learning capabilities. RL is based on the Markov decision process (MDP), which is a sequential decision mathematical model that has natural similarity to the TSP. Therefore, many recent algorithms have adopted RL for TSP, and they can be divided into three categories.

1. Deep learning algorithm combined with RL. The deep learning (DL) algorithm combined with RL exploits the perception ability of DL and the decision-making ability of RL. Its fast solution speed and strong generalization ability give this combination great potential in finding approximate TSP solutions. For example, Vinyals et al. (2015) [11] used supervised learning for training in a pointer network for TSP. Bello et al. (2016) [12] used an actor–critic policy gradient method in a recurrent neural network. Dai et al. (2017) [13] proposed an S2V-DQN algorithm using a graph embedding network trained by deep Q-learning. Deudon et al. (2018) [14] trained a neural network for TSP by policy gradient using the reinforcement learning rule with a critic. Ma et al. (2019) [15] used hierarchical RL for training in a graph pointer network for TSP. Stohy et al. (2021) [16] used an actor-critic method for training in a hybrid pointer network. These algorithms use RL to train neural networks by testing a large number of small-scale TSP datasets, which consumes a great deal of time and resources, and the accuracy of the solution is not ideal. The essence of this type of algorithm is deep learning, using RL only as a training method.
2. Heuristic algorithm combined with RL. The heuristic algorithm combined with RL uses the strong generalization ability of RL to improve the heuristic algorithm to obtain better and faster solutions. For example, Liu et al. (2009) [17] proposed an improved GA algorithm with reinforcement mutation, using the genetic algorithm as the framework and reinforcement learning as a mutation operator, making it essentially a heuristic algorithm. Alipour et al. (2018) [18] proposed a hybridization of GAs and a multi-agent reinforcement learning (MARL) heuristic, which uses GA as a tour improvement heuristic and MARL as a construction heuristic. Costa et al. (2020) [19] proposed a 2-opt heuristic algorithm combined with deep RL for TSP, which essentially enhances the learning process of 2-opt. Combining the advantages of Q-learning (QL), Sarsa, and the Monte Carlo algorithm, Zheng et al. (2020) [20] proposed a variable strategy reinforced (VSR) approach, optimized the k-opt process of LKH based on this, and designed VSR-LKH for TSP. Optimizing the parameters of the biased random-key genetic algorithm (BRKGA) by QL, Chaves et al. (2021) [21] proposed a BRKGA-QL algorithm for TSP. These algorithms are still heuristic, using RL only to optimize parameters or strategies.
3. RL algorithm. The above two categories of algorithms are not RL; they use RL for training or optimization. Algorithms based on RL are less common. For example, Ottoni et al. (2018) [22] proposed two RL algorithms for TSP, the QL and Sarsa (state action reward state action) algorithms, where the RL parameter estimation is based on a response surface model (RSM). The algorithm only optimizes the parameters of RL, and does not improve the algorithm framework, so the results are worse than the above two types of algorithms. For this reason, we design the improved QL-based algorithms in this paper to find a better QL framework to increase the efficiency of this kind of algorithm and provide a new way to solve the TSP. The principal contributions are as follows:

We introduce an improved QL algorithm for solving the TSP. A new reward matrix is constructed that is able to evaluate the immediate reward of the action more accurately compared to the original reward matrix. To reduce the size of the initial search space, a new initial Q-table is constructed. To balance exploration and exploitation, a new action selection strategy is designed. To more accurately assess the actions of the agent, a new Q-value update formula is designed. A local search strategy is also designed to enhance the algorithm’s local optimization capability.

The remainder of this paper is organized as follows. Section 2 presents the basic theoretical concept of RL, and a QL algorithm for the TSP problem is constructed. After analysis of the algorithm, some improvements are examined. In section 3.1, based on four proposed improvement strategies, an adaptive beam search QL algorithm is constructed, and a dynamic sub-route-based adaptive beam search QL algorithm is studied in section 3.2 to further improve performance of the algorithm. Section 4 discusses our experiments. Section 5 presents conclusions and suggested future work.

## 2. Principle and methodology of QL algorithm to solve TSP

### 2.1. Theoretical foundation

RL is an important machine-learning method proposed by Minsky in 1956. Its model is based on MDP, which shows significant advantage and potential in sequential decision problems. Its powerful exploration and autonomous learning abilities [23] are widely used in game-playing [24–26], robotic control [27, 28], and transportation [29–31].

As a methodological framework for learning, prediction, and decision-making, the agent learns the optimal policy by interacting with the environment [32]. Based on different ways of action selection, RL can be categorized as value-based, policy-based, or actor–critic learning. QL and Sarsa are two representative value-based RL algorithms. Considering that the value-based algorithm is applicable to a discrete action space, QL requires fewer parameters and has a better exploration ability [33], and is likely to find a better solution than Sarsa; thus, we focus on QL algorithms for TSP.

As a model-free RL algorithm, in QL, the agent continually interacts with the environment to obtain rewards, and the action-selection strategy can be evaluated and optimized to maximize the cumulative reward [34–36].

The QL algorithm can be described as an MDP, which is usually represented by a five-tuple, <*S*, *A*, *P*, *R*, γ> [37]. *S* is the state of the environment, *s* (*s*∈*S*) represents a certain state, and the environment is the learning object in which the agent performs actions to change its state. *A* is the action set of the agent, and *a* (*a*∈*A*) represents a certain action. As a learner, the agent tries all possible actions to cognize the environment to explore the proper actions. *P* is a matrix of state transition probabilities, and *p*(*s*,*a*)∈*P* is the probability that the agent performs action *a* under state *s*, and the selection of action is determined by policy *π*. *R* is the reward matrix, and *r*(*s*,*a*)∈*R* is the immediate reward obtained by the agent after performing action *a* in status *s*. *γ* is the discount factor that determines the ratio between the immediate and cumulative rewards, whose higher value indicates a greater emphasis on the latter.

QL utilizes the Q-table and reward matrix *R* to respectively evaluate the cumulative and immediate rewards of actions. The Q-value is updated as follows. Given state of the environment *s*_*t*_ of step *t*, the agent selects action *a*_*t*_ under the *ε-greedy* strategy to affect the environment so that the state is transferred to *s*_*t+1*_, and an immediate reward *r*(*s*_*t*_,*a*_*t*_) is obtained. Then, the Q-value is updated as Formula (1).

$$q(s_{t},a_{t})=(1-\alpha )*q(s_{t},a_{t})+\alpha *\left(r\left(s_{t},a_{t}\right)+\gamma *\underset{a_{t+1}\in A}{max}q\left(s_{t+1},a_{t+1}\right)\right)$$ (1)

where *q*(*s*_t_,*a*_*t*_) is the cumulative reward of taking action *a*_*t*_ in state *s*_*t*_; $\underset{a_{t+1}\in A}{max}q(s_{t+1},a_{t+1})$ is the maximum cumulative reward that can be obtained by taking action *a*_*t+1*_ in state *s*_*t+1*_; *α* is a learning factor, *α*∈[0,1]; and γ is a discount factor, *γ*∈[0,1].

The *ε-greedy* strategy guides the agent to choose the action with the largest Q-value with probability *ε* and to randomly select the other action with probability 1−*ε*.

The pseudocode of QL is shown as Algorithm 1.

Algorithm 1. QL

1. Begin

2. Construct the tuple;

3.     Initialize Q-table and reward matrix *R*;

4.     Define parameters *α*, *γ*, *ε*;

5.     Initialize the maximum number of iterations *I*_*max*_;

6.     while (current number of iterations< *I*_*max*_)

7.       Initialize *t* and *s*_*t*_;

8.       While (*s*_*t*_ is not the terminal state)

9.         Select action *a*_*t*_ according to *ε-greedy* policy in state *s*_*t*_;

10.         Calculate the immediate reward *r(s*_*t*_,*a*_*t*_*)*;

11.         Update *q(s*_*t*_,*a*_*t*_*)* according to Formula (1);

12.         *t* = *t* + 1;

13.       end while

14.     end while

15.     Obtain optimal policy according to Q-table.

16.End

### 2.2. Problem mapping of QL algorithm for TSP

Solving the TSP using the QL algorithm requires the construction of five-tuples, with the following parameters.

*n*: total number of cities;

*t*: number of visited cities, 1≤*t*≤*n*;

*S*_*t*_: set of visited cities after visiting *t* cities;

*s*_*t*_: number of *t*-th visited city, *s*_*t*_∈*S*_*t*_;

*A*_*t*_: set of unvisited cities after visiting *t* cities;

*a*_*t*_: number of next city to be visited after city *s*_*t*_, *a*_*t*_∈*A*_*t*_;

*i*: number of current city, 1≤*i*≤*n*, which is used as the row value of the matrix *P*, *R*, *Q*, *D*;

*j*: number of next city to be visited from current city, 1≤*j*≤*n*, which is used as the column value of the matrix *P*, *R*, *Q*, *D*;

*I*_*c*_: current iteration number;

*I*_*max*_: maximum iteration number;

*P*: action selection probability matrix,

P=[p(1,1)⋯p(1,n)⋮⋱p(i,j)⋱⋮p(n,1)⋯p(n,n)],

where *p*(*i*,*j*) is the probability of visiting city *j* from city *i*;

*R*: reward matrix, also known as the immediate reward matrix,

R=[r(1,1)⋯r(1,n)⋮⋱r(i,j)⋱⋮r(n,1)⋯r(n,n)],

where *r*(*i*,*j*) is the immediate reward obtained by visiting city *j* from city *I*;

*Q*: Q-table, also known as the cumulative reward matrix,

Q=[q(1,1)⋯q(1,n)⋮⋱q(i,j)⋱⋮q(n,1)⋯q(n,n)],

where *q*(*i*,*j*) is the cumulative reward obtained by visiting city *j* from city *i*;

*D*: distance matrix,

D=[d(1,1)⋯d(1,n)⋮⋱d(i,j)⋱⋮d(n,1)⋯d(n,n)],

where *d*(*i*,*j*) is the distance from city *i* to city *j*.

### 2.3. Construction of proposed QL algorithm

The process of the algorithm for solving the TSP can be described as follows. Starting from city *s*_*t*_ (the initial value of *t* is 1), select city *a*_*t*_ from *A*_*t*_ and visit it according to the *ε-greedy* strategy, determine the immediate and cumulative rewards from *s*_*t*_ to *a*_*t*_. Repeat this process until all cities are visited.

(1) Construct reward matrix *R*, where each element is calculated as

$$r(i,j)=\frac{1}{d(i,j)};$$ (2)

(2) Construct initial Q-table, where each element is calculated as

q(i,j)=0; (3)

(3) Determine the *ε-greedy* strategy as

$$p(s_{t},a_{t})=\left\{ \begin{array}{l} \varepsilon ,\;if\;q\left(s_{t},a_{t}\right)=\underset{j\in A_{t}}{\max}q(s_{t},j)\\ \frac{1-\varepsilon }{n-t-1},\;otherwise \end{array}\right.$$ (4)

Formula (4) shows that we start from city *s*_*t*_ and visit city *a*_*t*_ with a certain probability. The city with the largest Q-value has a probability of *ε* to be visited, while the other (*n−t−*1) cities have a probability of (1−*ε*) /(*n−t−*1) to be visited.

The pseudocode of the QL algorithm for the TSP is shown as Algorithm 2.

Algorithm 2. QL algorithm for TSP

1. Input: TSP instance with *n* cities

2. Output: optimal solution

3. Begin

4.     Initialize the *Q* according to Formula (3);

5.     *ε* = 0.95;

6.     *I*_*c*_ = 0;

7.     while (*I*_*c*_ < *I*_*max*_)

8.       Randomly select a city to be the initial one and denoted it as *s*_*1*_;

9.       *S*_*1*_ = {s_1_};

10.       *A*_*1*_ = {*a*_*1*_, *a*_*2*_, …, *a*_*n-1*_};

11.       *t* = 1;

12.       while (*t* ≤ *n)*

13.         Determine the next city *a*_*t*_ to be visited from *s*_*t*_ according to Formula (4);

14.         Calculate the immediate reward *r*(*s*_*t*_,*a*_*t*_) from city *s*_*t*_ to *a*_*t*_ according to Formula (2);

15.         Update the cumulative reward *q*(*s*_*t*_,*a*_*t*_) from city *s*_*t*_ to *a*_*t*_ according to Formula (1);

16.         *A*_*t+1*_ = *A*_*t*_-{*a*_*t*_};

17.         *s*_*t+1*_ = *a*_*t*_;

18.         *S*_*t+1*_ = *S*_*t*_∪{*s*_*t+1*_};

19.         *t* = *t* + 1;

20.         end while

21.         Calculate and save the length of *n* cities access sequence in set *S*_*n*_;

22.         *I*_*c*_ = *I*_*c*_ + 1;

23.       end while

24.       The route corresponding to *S*_*n*_ with the shortest route length will be the optimal one.

25.End

### 2.4. Analysis of results

We selected 18 instances of 30–1655 cities in TSPLIB to test the proposed QL algorithm. The parameters *α*, *γ*, and *ε* of the algorithm were 0.1, 0.1, and 0.95, respectively. The results are compared with those of the VDWOA and DWOA algorithms [9] in the same environment. As only 12 instances are listed in [9], results of the six other instances were tested by VDWOA and DWOA; the details are listed in Table 2.

The results are as follows. For instances with fewer than 500 cities, the QL algorithm is 2–40 times faster than the comparative algorithms; for instances with 500–1000 cities, it is 4–60 times faster, and for instances with more than 1000 cities, it is 5–40 times faster. The QL algorithm has distinct advantages in solution time, but the accuracy is relatively poor.

The proposed QL algorithm (Algorithm 2) is only a simple simulation of the traditional QL algorithm (Algorithm 1), which does not consider the characteristics of TSP and the defects of the QL algorithm itself. There are four reasons for its poor accuracy:

1. Formula (3) for calculating elements of reward matrix *R* only considers the distance between the current city *i* and city *j*, the next to be visited, without considering the impact of the distance between city *j* and subsequent cities. This is too greedy, and can lead to a high probability of selecting the local optimum city.
2. The Q-table is simply initialized with 0, giving all cities the same initial Q-value, so that the algorithm searches nearly blindly in the early stage, resulting in a large search range and decreasing the convergence speed. Due to the great randomness of city selection in the early stage, the probability of worse cities being selected may be increased, causing the algorithm to easily fall into local optima.
3. Cities are selected based on a *ε-greedy* strategy. If *ε* is too large, the selection probability of the global optimal city will be reduced, and the search scope smaller, causing premature convergence. If *ε* is too small, the probability of selecting a globally worse city will increase, and the search scope of the algorithm will be larger, causing difficult convergence.
4. There is no new city to visit after the last city, and all that remains is to return to the starting city. Thus, there is no need to consider the cumulative reward, and Formula (1) is no longer suitable for updating the Q-value.

## 3. QL-based algorithms for TSP

We studied several methods to improve the QL algorithm based on the above analysis.

### 3.1. Self-adaptive beam search QL algorithm

We propose a self-adaptive beam search QL algorithm, ABSQL. Four strategies are introduced: the weighting function-based reward matrix, power function-based initial Q-table, self-adaptive *ε-beam* search strategy, and a new formula for updating the Q-value.

#### 3.1.1. Weighting function-based reward matrix

For reason (1) above, we draw on the idea of the finite discount cumulative reward strategy [38]. Based on Formula (2), we introduce *d*_*min*_ and *d*_*avg*_ to consider the effect of the distance from city *j* to subsequent cities from local and global dimensions, respectively, and compute

$$r´(i,j)=\theta_{1}*r(i,j)+\frac{{(1-\theta_{1})*\theta }_{2}}{d_{min}}*\frac{\left(1-\theta_{1})*(1-\theta_{2}\right)}{d_{avg}},$$ (5)

where *θ*_*1*_ and *θ*_*2*_ control the influence degree of city i on city j and its successor, respectively, *θ*_*1*_∈(0.5,1), *θ*_*2*_∈(0.5,1); and *d*_*min*_ and *d*_*avg*_ are the minimum and average distances, respectively, from city *j* to the other cities to be visited.

The above strategy (denoted as *stgy_1*) synthetically considers the minimum and average distance between the current city to be visited and subsequent cities. *stgy_1* can mitigate the greediness of the original reward matrix and increase the accuracy of rewards to find a higher-quality solution.

#### 3.1.2. Power function-based initial Q-table

For reason (2) above, since the distances between cities will affect city selection, the reward matrix constructed according to Formula (5) will positively affect the selection of cities. Therefore, we introduce a power function to cause positive correlation between the initial Q-table and the improved reward matrix. The improved initial Q-table is constructed as

$$q^{\prime (i,j)}=r^{\prime }{\left(i,j\right)}^{\delta },$$ (6)

where *δ* is a parameter to control the correlation between *q*(*i*,*j*) and *r´*(*i*,*j*), *δ*∈(1.0,1.2).

The improved initial Q-table (denoted as *stgy_2*) can reduce the search scope and enhance the search ability in the early stage, enabling faster convergence and a better-quality solution.

#### 3.1.3. Self-adaptive *ε-beam* search strategy

For reason (3) above, considering that the beam search algorithm has the characteristics of fast operation and a tendency to avoid local optima, a self-adaptive *ε-beam* search strategy is designed. By improving the fixed *ε* to be self-adaptive and replacing the greedy search with a beam search, the probability of action selection can be properly controlled.

(1) Self-adaptive *ε* strategy

In the early stage of the algorithm, the search behavior should be relatively random and the search scope should be large. In this stage, the algorithm focuses on exploration to avoid falling into local optima. In the later stage, the search scope should be small, and the algorithm turns to exploitation, while the convergence speed is gradually accelerated. Exploration and exploitation can be controlled by adjusting the value of *ε*. With a larger value, the algorithm tends more toward exploitation. Therefore, the value of *ε* should increase with iteration. The improved calculation is

$$\varepsilon ´=b+(c-b)*\sqrt{\frac{I_{c}}{I_{max}}},$$ (7)

where *b* and *c* are parameters to control the respective initial and final values of *ε*.

(2) Beam search strategy

Beam search is a heuristic graph search algorithm [39–41] that will gradually cut off some poor-quality nodes and reserve some high-quality nodes. It is usually adopted when the solution space of the graph is relatively large, to decrease the space and time cost of the search.

Based on a beam search, we sort Q-values of unvisited cities in descending order, and then select the top several as the next candidates to be visited. Considering the time cost and complexity of the algorithm, the number of candidate cities is set to 2, i.e., the current optimal or the second optimal city is selected as the next visited city. Compared with the greedy strategy, which only keeps the current optimal city, it has a larger search scope, which can increase the possibility of selecting the global optimal city, and makes the algorithm less likely to fall into local optima.

In summary, the self-adaptive *ε-beam* search strategy (denoted as *stgy**_**3*) can be described as

$$p´(s_{t},a_{t})=\left\{ \begin{array}{l} \varepsilon ´,\;q´(s_{t},a_{t})=\underset{j\in A_{t}}{\max}\;q´(s_{t},j)\;(a)\\ \left(1-\varepsilon ´)*\varepsilon ´,\;q´(s_{t},a_{t})=\underset{j\in A_{t}}{\mathrm{submax}}\;q´(s_{t},j)\;(b\right)\\ \frac{{\left(1-\varepsilon ´\right)}^{2}}{n-t-2},\;\mathrm{other}\;(c) \end{array}\right.$$ (8)

where formula (a) indicates that starting from city *s*_*t*_, the city with the maximum Q-value is selected with probability *ε´*, formula (b) indicates that the city with the sub-maximum Q-value is selected with probability (1-*ε´*) * *ε´*, and formula (c) indicates that other cities are selected with probability (1-*ε´*)^2^/(*n*-*t*-2) in *A*_*t*_.

The improved strategy can balance exploration and exploitation, expand the search scope in the early stage, and enhance the search ability in the middle and later stages.

#### 3.1.4. Improvement of Q-value update formula

For reason (4) above, the Q-value of the last visited city does not contain long-term rewards, so the value calculated by using Formula (1) that contains long-term rewards is inevitably higher than its real Q-value. Furthermore, the influence of such overestimation will gradually accumulate with iteration, making the estimation of the Q-table more inaccurate, affecting the judgment of city selection. Therefore, Formula (1) is no longer suitable for updating the Q-value of the last visited city.

The last visited city has no other city to visit and needs only to return to the starting city; hence the long-term reward in Formula (1) should not be considered. The improved Q-value update formula is

$$q´(s_{t},a_{t})=\left\{ \begin{array}{l} \left(1-\alpha )*q´(s_{t},a_{t})+\alpha *(r´(s_{t},a_{t})+\gamma *\underset{a_{t+1}\in A}{\max}q´(s_{t+1},a_{t+1}))\;(a\right)\\ \left(1-\alpha )*q´(s_{t},a_{t})+\alpha *r´(s_{t},a_{t}),\;(b\right) \end{array}\right.$$ (9)

where formula (b) is used for the last visited city, and formula (a) for all other cities.

The improved Q-value update formula (denoted as *stgy_4*) can make the values of the Q-table more accurate when evaluating city selection.

The pseudocode of the ABSQL algorithm is presented as Algorithm 3.

Algorithm 3. ABSQL algorithm for TSP

1. Input: TSP instance with *n* cities

2. Output: optimal solution

3. Begin

4.     Initialize the Q table according to Formula (6);

5.     *I*_*c*_ = 0;

6.     while (*I*_*c*_ < *I*_*max*_)

7.       Calculate *ε´* according to Formula (7);

8.       Select one city randomly to be the starting one and denoted it as *s*_*1*_;

9.       Let *S*_*1*_ *= {s*_*1*_*}*, *A*_*1*_ *= {a*_*1*_, *a*_*2*_, *…*, *a*_*n-1*_*}* and the number of cities visited *t* = 1;

10.       while (*t*≤*n*)

11.         Determine the next city *a*_*t*_ to be visited from *s*_*t*_ according to Formula (8);

12.         Calculate the immediate return value *r´*(*s*_*t*_,*a*_*t*_) according to Formula (5);

13.         if (*t*! = *n*)

14.           Update the cumulative reward *q´*(*s*_*t*_,*a*_*t*_) from city *s*_*t*_ to *a*_*t*_ according to Formula (9a);

15.         else if (*t* = = *n*)

16.           Update the cumulative reward *q´*(*s*_*t*_,*a*_*t*_) from city *s*_*t*_ to *a*_*t*_ according to Formula (9b);

17.         end if

18.         *A*_*t+1*_ *= A*_*t*_*-{a*_*t*_*}*;

19.         *s*_*t+1*_ *= a*_*t*_;

20.         *S*_*t+1*_ *= S*_*t*_∪*{s*_*t+1*_*}*;

21.         *t = t* + 1;

22.       end while

23.       Calculate and save the length of *n* cities access sequence in set *S*_*n*_;

24.       *I*_*c*_ = *I*_*c*_ + 1;

25.     end while

26.     The route corresponding to *S*_*n*_ with the shortest route length will be the optimal one.

27.End

#### 3.1.5. Analysis of results

We used the 18 instances in Section 2.4 to test the effectiveness of the proposed ABSQL algorithm through analysis of the length and graph of the route. The parameters *α* and *γ* used in the ABSQL algorithm are the same as in the QL algorithm. The newly added parameters *θ*_*1*_, *θ*_*2*_, *δ*, *b* and *c* are 0.8, 0.8, 1.12, 0.25, and 1.05, respectively.

(1) Route length analysis

We compare solutions obtained by the ABSQL algorithm with those of QL and VDWOA, with results as shown in Table 2. Denote *min*, *avg*, and *max* to be the minimum, average, and maximum values, respectively, with deviation rates

$$S_{min}=\frac{min-opt}{opt}\times 100\%$$ (10)

$$S_{avg}=\frac{avg-opt}{opt}\times 100\%$$ (11)

$$S_{max}=\frac{max-opt}{opt}\times 100\%,$$ (12)

where *opt* is the known optimal solution.

The comparison of ABSQL and QL shows that ABSQL has a faster solution, the quality of which is greatly improved.

For the 18 instances, *min* of ABSQL is better than that of QL, and its *S*_*min*_ is 0–92% less than that of QL. Comparing ABSQL and VDWOA, in terms of time, ABSQL exceeds VDWOA in all instances. In terms of solution quality, the following results are obtained.

For 14 small-scale instances with the total number of cities less than 500, the *min* of ABSQL is slightly worse than that of VDWOA for eight instances, and its *S*_*min*_ is 0.08%–5.7% greater. However, the *avg* of ABSQL has clear advantages over VDWOA in 10 instances, and its *S*_*avg*_ is 0.31%–7.25% less. For four medium- and large-scale instances with more than 500 cities, the *min* of ABSQL is better than that of VDWOA, and its *S*_*min*_ is 0.67%–5.11% less. Since VDWOA takes too long for medium- and large-scale instances, it cannot obtain enough solutions in a limited time to calculate *avg*. Therefore, the *avg* and *S*_*avg*_ of the two algorithms are not compared.

The above analysis shows that ABSQL has a better ability than VDWOA for medium- and large-scale instances. However, as comparing *opt* values reveals, ABSQL still has some deficiencies. For small-scale instances, the *S*_*min*_ of ABSQL is 0–10.95%, and for medium- and large-scale instances, the *S*_*min*_ of ABSQL is 12.34%–14.31%, i.e., the *S*_*min*_ of ABSQL increases with the scale of the problem, so the solution accuracy of ABSQL requires further improvement.

(2) Route diagram analysis

**Fig 1:**
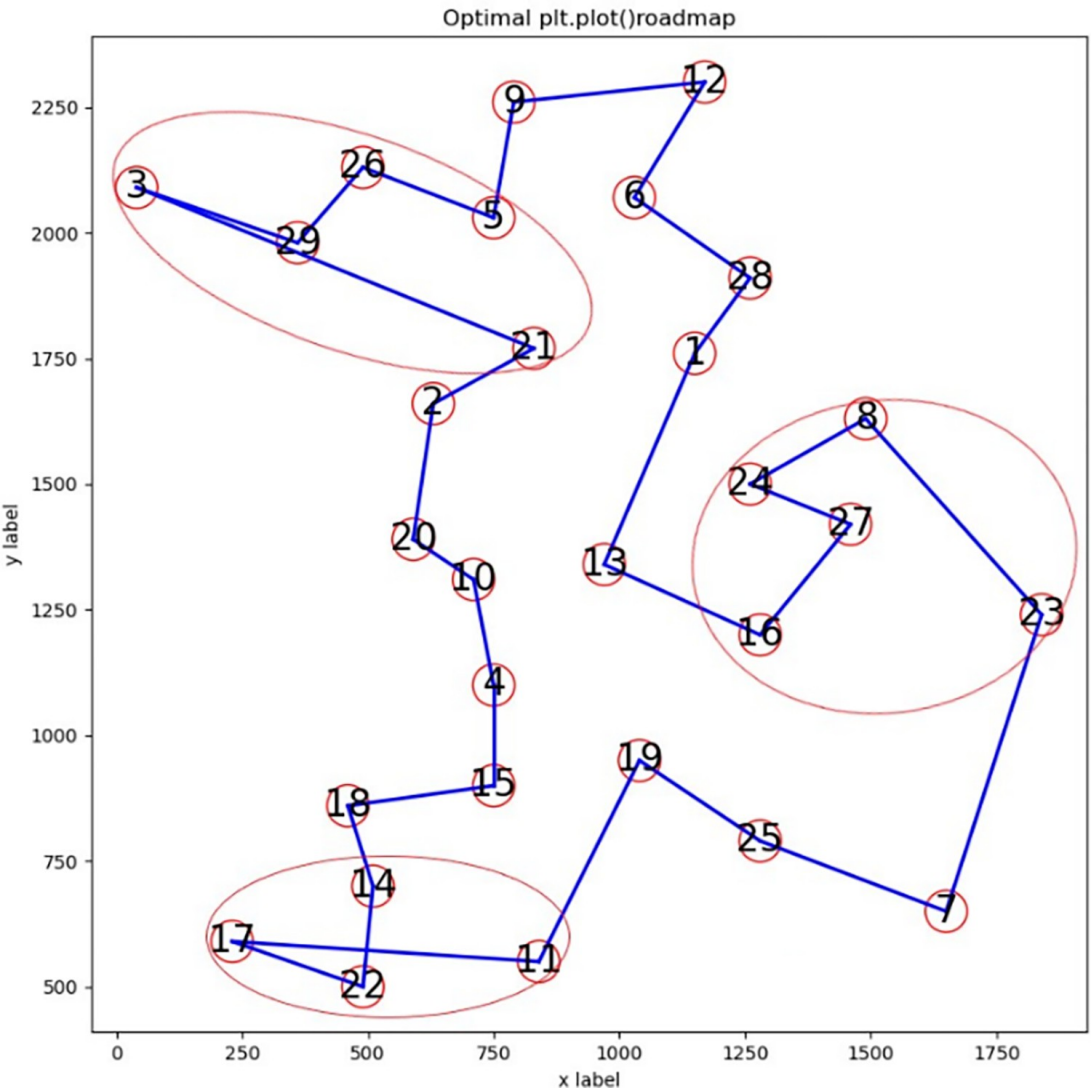
Route for bays29 output by ABSQL algorithm.

**Fig 2:**
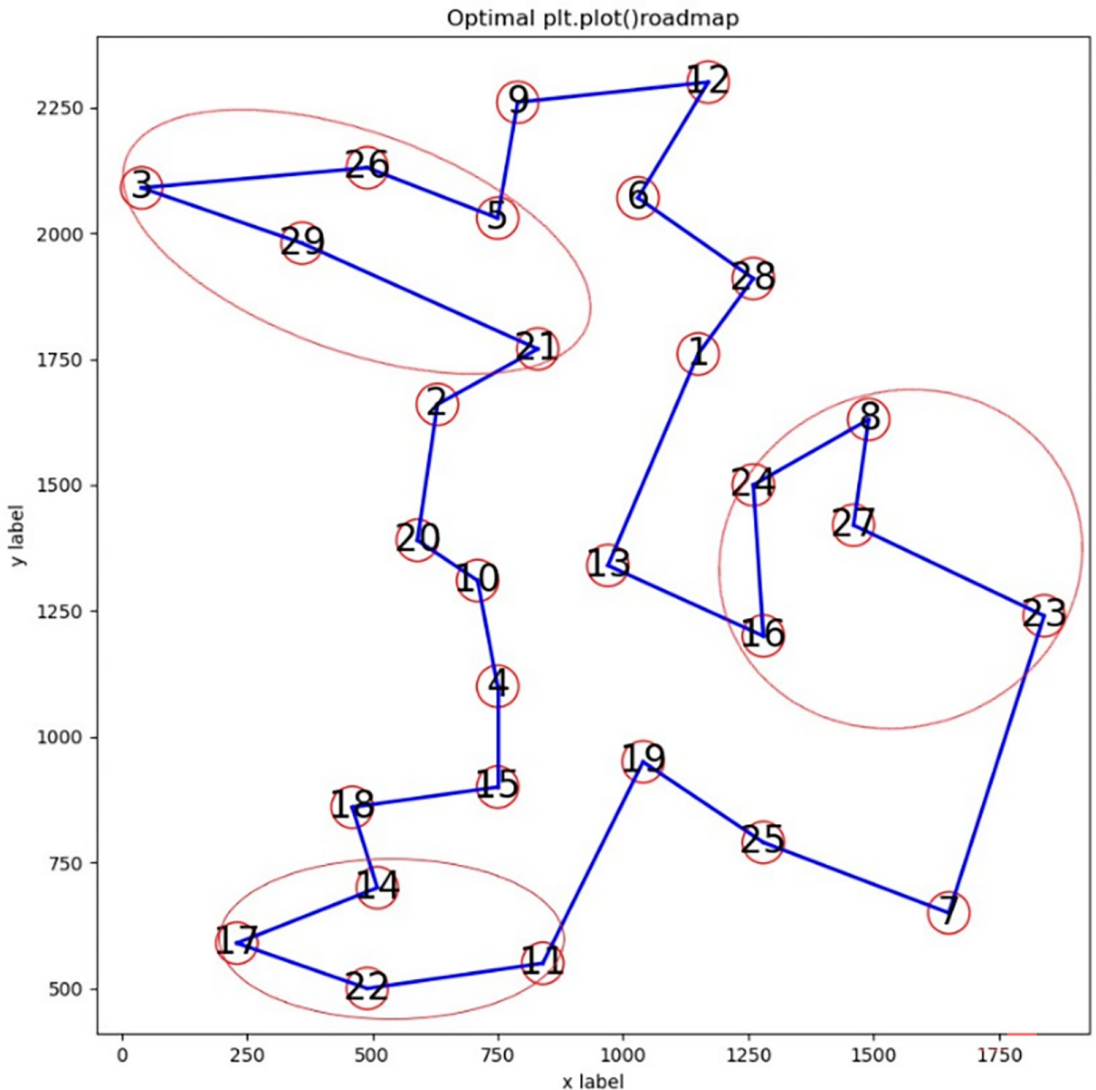
Optimal route of bays29.

Analyzing the route diagrams obtained from the above instances, a common problem is found: one or more sub-routes are not completely optimized. We select instance bays29 to analyze this problem from two aspects. Fig 1 is an incompletely optimized route diagram, and Fig 2 is an optimal route diagram. The X- and Y-axes represent the abscissa and ordinate, respectively, of the city; circled numbers indicate serial numbers of cities; solid lines are routes between cities. The three incompletely optimized sub-routes in Fig 1 and their corresponding fully-optimized sub-routes in Fig 2 are marked by oval circles, respectively.

1) Cross or overlap between edges

In Fig 1, sub-route 11-17-22-14 is incompletely optimized, with a crossover between edges (11,17) and (22,14). In Fig 2, there is no crossover of edges, sub-route 11-22-17-14 is fully optimized, and sub-route 11-17-22-14 can be optimized to 11-22-17-14 by swapping nodes 17 and 22.

2) No cross or overlap between edges

In Fig 1, there is no crossover or overlap between edges of sub-route 23-8-24-27-16. However, the sub-route is not completely optimized. This is mainly because the sum of the lengths of edges (23,27) and (27,8) is less than that of (16,27) and (27,24). Sub-route 23-27-8-24-16 of Fig 2 is optimal. Sub-route 23-8-24-27-16 can be optimized to 23-27-8-24-16 by swapping nodes 8 and 24, and then nodes 24 and 27. Similarly, sub-route 21-3-29-26 of Fig 1 can be improved to the better sub-route 21-29-3-26 of Fig 2 by swapping nodes 3 and 29.

Through the above analysis, it can be seen that the incomplete optimization of sub-routes is the main reason for the relatively poor solution of the ABSQL algorithm compared with the known optimum. Considering that the route length can be shortened by swapping some nodes of the sub-route, we introduce the *2-opt* strategy to further optimize the sub-route.

### 3.2. Dynamic sub-route-based ABSQL algorithm

To solve the problem of incomplete optimization of sub-routes, static and dynamic sub-route optimization strategies are designed to optimize routes output by ABSQL.

#### 3.2.1. Static sub-route optimization

In this section, we perform following operations. Denote the route output by ABSQL as *S*_*n*_. A partition operation is performed first on *S*_*n*_, as follows. Starting from the first node, successively divide *S*_*n*_ (which has *n* nodes) into ⌊*n/m*⌋ sub-routes (each with *m* nodes), and denote these as *sr*_*1*_, *sr*_*2*_*… sr*_⌊*n/m*⌋_. If there is a sub-route with fewer than *m* nodes, it is denoted as *rp*, and the set of all sub-routes is *Sr* = {*sr*_*1*_, *sr*_*2*_, *…*, *sr*_⌊*n/m*⌋_, *rp*}. Because the number of nodes in each sub-route is fixed, the partition process is called static sub-route optimization. For sub-routes with four or more nodes in *Sr*, all nodes except the first and last are selected to perform 2-opt optimization, and the optimized set is denoted as *Sr*^*1*^. The optimization process is as follows.

Step 1: Select the second node in the sub-route as the starting node, as well as other nodes for (*m−*2) *(*m−*3)/2 instances of 2-opt optimization;

Step 2: If the length of the sub-route does not decrease after 2-opt optimization, the optimization of the sub-route is stopped; otherwise, the new sub-route is reserved and we return to step 1.

#### 3.2.2. Dynamic sub-route optimization

Since static sub-route optimization does not consider optimization of the first and last nodes of a sub-route, and the number of nodes on each sub-route is fixed, a dynamic optimization strategy is introduced in which the first and last nodes are considered at the same time. Dynamic optimization is performed based on *Sr*^*1*^ obtained by static optimization, so that the number of nodes on each sub-route can change over the optimization process, which is as follows.

Step 1: Suppose the number of current optimization rounds is *h*, with initial value 1, *h*≤*L*. The set of sub-routes after the *h-th* round of optimization is *Sr*^*h*^ = {*sr*_*1*_^*h*^, *sr*_*2*_^*h*^, …, *sr*_*f(h)-1*_^*h*^, *rp*^*h*^}, where *sr*_*1*_^*h*^ to *rp*^*h*^ represent the first to *f*(*h*)*-*th sub-routes, respectively; *f*(*h*) is calculated according to Formula (13), and *L* is the total number of optimization rounds, as determined by Formula (14).


$$f(h)=\lceil \frac{n}{m*2^{h-1}}\rceil$$ (13)

$$L=\left\{ \begin{array}{l} \lceil \frac{n}{200}\rceil ,\;n\leq 500\\ 3,\;500\leq n<1000\\ 4,\;n\geq 1000 \end{array}\right.$$ (14)

Step 2: All sub-routes in set *Sr*^*h*^ except for *rp*^*h*^ are merged in pairs in order, and if *sr*_*f(h)-1*_^*h*^ is left, it is merged with *rp*^*h*^;

Step 3: For each sub-route obtained in step 2, select nodes at 1/4 to 3/4 positions of the node sequence, and perform 2-opt optimization to obtain set *Sr*^*h+1*^;

Step 4: If *h*≤L, then let *h = h+1*, and return to step 2; otherwise, merge all sub-routes in *Sr*^*L+1*^ to obtain the new path.

By combining ABSQL with the dynamic sub-route optimization strategy (denoted as *stgy_5*), a dynamic sub-route-based self-adaptive beam search Q-learning algorithm (DSRABSQL) for TSP is designed.

The pseudocode of the above algorithm is shown as Algorithm 4.

Algorithm 4. DSRABSQL algorithm for TSP

1. Input: *S*_*n*_ of ABSQL algorithm

2. Output: optimal solution

3. Begin

4.     Initialize *m*;

5.     *S*_*n*_ obtains *Sr* according to the division operation in Section 3.4.1

6.     Optimize *Sr* according to step 1 and step 2 in Section 3.4.1 to get *Sr*^*1*^

7.     Initialize *L* according to Formula (14);

8.     Initialize *h* = 1;

9.     while (*h* ≤ *L*)

10.         Execute step 2 and step 3 of section 3.4.2

11.         *h* = *h* + 1;

12.     end while

13.         Merge all sub-routes in *Sr*^*L+1*^ in order to obtain an optimal route.

14.End

#### 3.2.3. Analysis of results

We used the 18 instances in Section 2.4 to test the proposed DSRABSQL algorithm. The parameters *α*, *γ*, *θ*_*1*_, *θ*_*2*_, *δ*, *b* and *c* used in the DSRABSQL algorithm are the same as in the ABSQL algorithm, with the added parameter *m* of 10. The performance of DSRABSQL is analyzed by comparing its optimal route diagrams with those of ABSQL.

Since most instances have too many cities or an uneven distribution, the route diagrams are not clear. We list the route diagrams of berlin52 and tsp225 for illustration.

**Fig 3:**
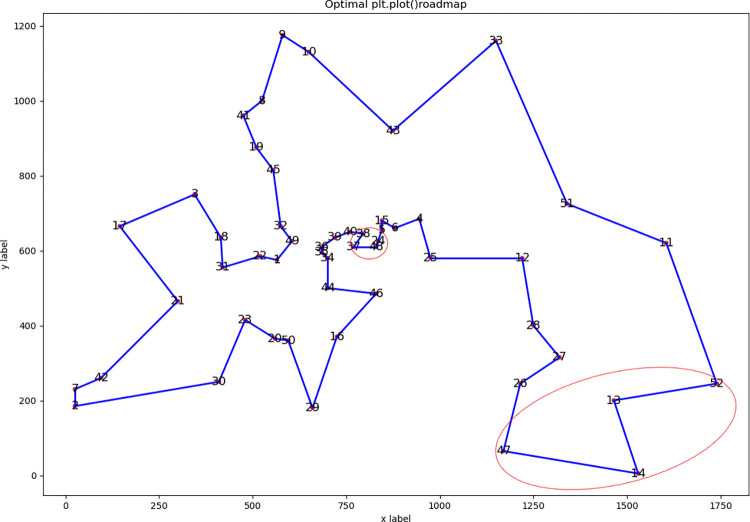
The optimal route of Berlin52 output by ABSQL algorithm.

**Fig 4:**
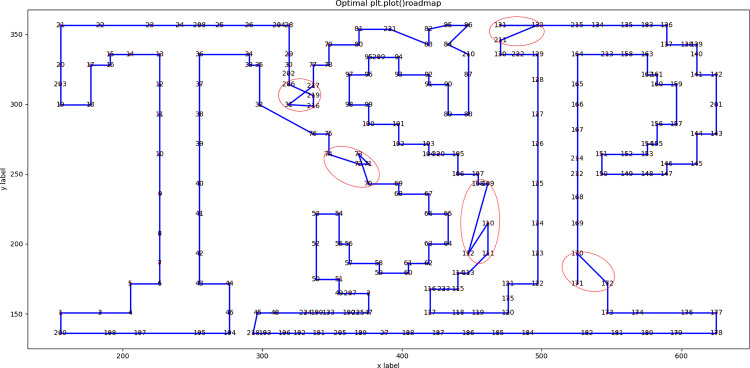
The optimal route of Tsp225 output by ABSQL algorithm.

**Fig 5:**
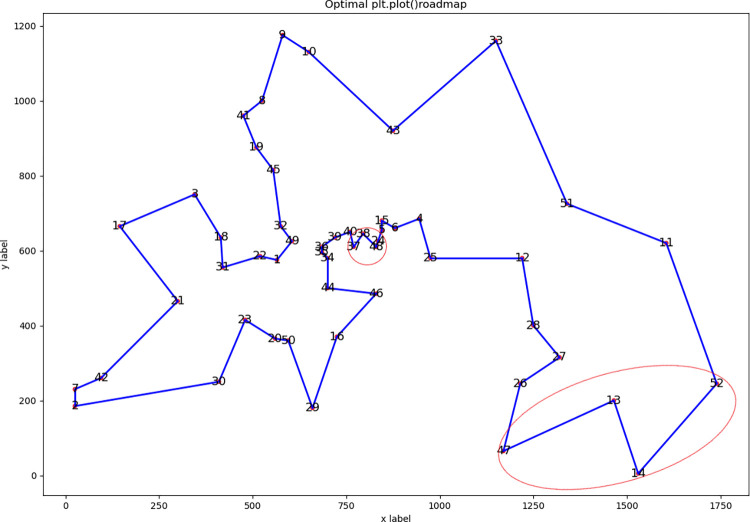
The optimal route of Berlin52 output by DSRABSQL algorithm.

**Fig 6:**
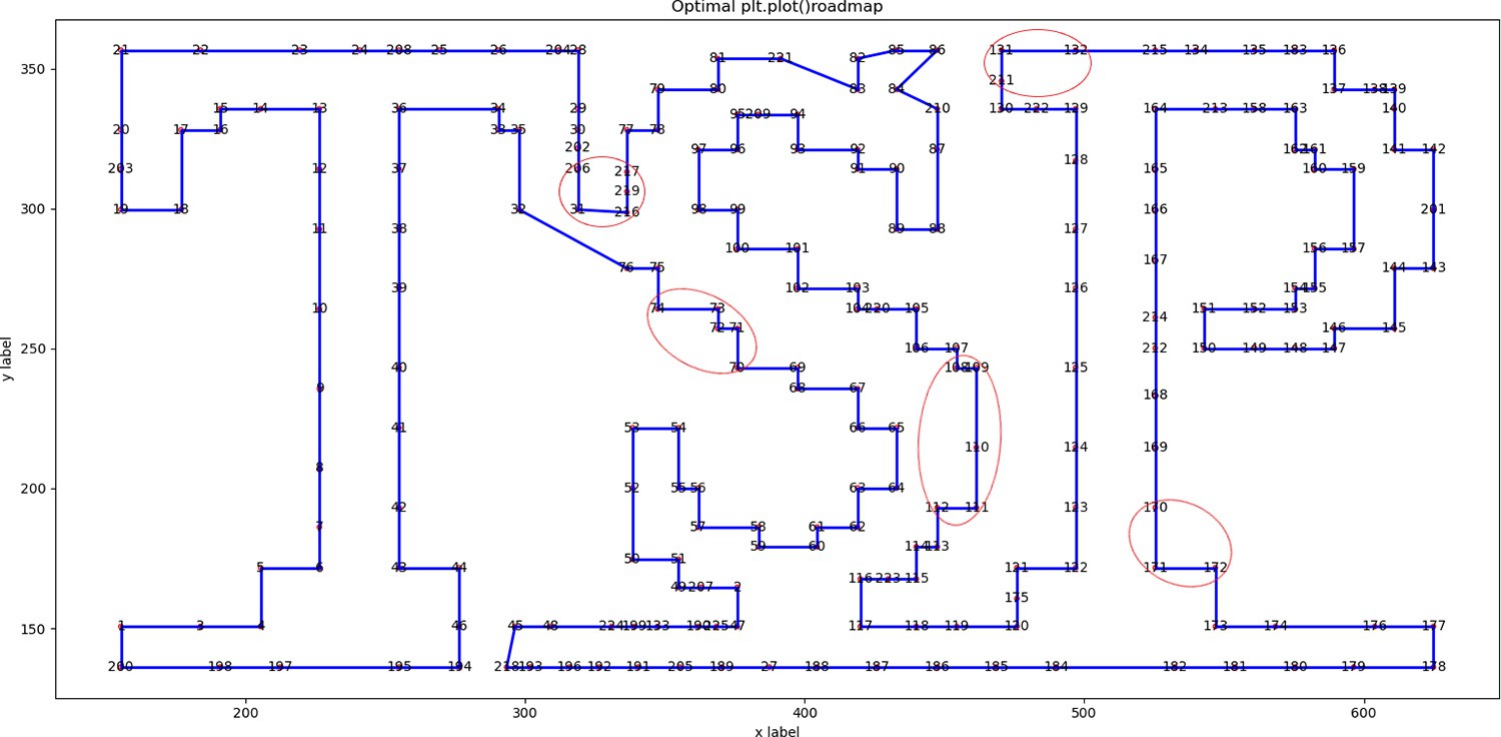
The optimal route of Tsp225 output by DSRABSQL algorithm.

Figs 3 and 4 are the optimal routes of berlin52 and tsp225, respectively, as obtained by ABSQL. Figs 5 and 6 are the corresponding routes as obtained by DSRABSQL.

From the analysis of the route diagram, it can be found that the sub-routes marked in Figs 3 and 4 are not completely optimized, and the sub-routes in the corresponding positions of Figs 5 and 6 are completely optimized. Therefore, by enhancing the local optimization ability of ABSQL, DSRABSQL greatly improves the solution accuracy of TSP.

## 4. Experiments and analysis of results

The simulation is carried out in Spyder and implemented on an Intel Core i5-7500 CPU at 3.4 GHz, with 8 GB memory and a Windows 10 (64-bit) operating system. We take 18 TSP instances and test them with city’s scale from 30 to 1655. The instances that used for this experiment is taken from TSPLIB (http://comopt.ifi.uni-heidelberg.de/software/TSPLIB95/) library. Table 1 lists the simulation parameters of the three algorithms to elaborate on the characteristics of the parameters adopted by each algorithm.

**Table 1:** Simulation parameters for the QL, ABSQL and DSRABSQL algorithms.

Parameter	Values		
	QL	ABSQL	DSRABSQL
*α* (formulas 1 and 9)	0.1	0.1	0.1
*γ* (formulas 1 and 9)	0.1	0.1	0.1
*ε* (formulas 4 and 8)	0.95	dynamic	dynamic
*θ*_*1*_ (formula 5)	—	0.8	0.8
*θ*_*2*_ (formula 5)	—	0.8	0.8
*δ* (formula 6)	—	1.12	1.12
*b* (formula 7)	—	0.25	0.25
*c* (formula 7)	—	1.05	1.05
*m* (formula 13)	—	—	10

The values of the parameters listed in Table 1 were obtained through experimental tests, and the specific procedures are as follows.

**Procedure 1:** Determine *α*, *γ* and *ε* of the QL algorithm.

1. Set *ε* based on QL-related books and literature;
2. Set the initial value, final value and step size of parameters *α* and *γ*, and select the optimal values of these parameters according to the test results.

**Procedure 2:** Determine *θ*_*1*_, *θ*_*2*_, *δ*, *b* and *c* of the ABSQL algorithm.

1. Set the initial values, final values and step sizes of parameters *θ*_*1*_, *θ*_*2*_ in *stgy*_*1*, parameter *δ* in *stgy*_*2*, and parameters *b* and *c* in *stgy*_*3* in succession, and select the optimal values of these parameters according to the test results;
2. The parameters *α* and *γ* in *stgy*_*4* inherit the optimal values of *α* and *γ* from the QL algorithm.

**Procedure 3:** Determine parameter *m* of the DSRABSQL algorithm.

Set the initial value, final value and step size of parameter *m* in *stgy*_*5*, and select the optimal value of these parameters based on the test results.

The QL, ABSQL and DSRABSQL algorithms are iterated for *I*_*max*_ times, respectively. The results are taken in *n*_*t*_ simulation runs for each instance. *I*_*max*_ and *n*_*t*_ are calculated as

$$I_{max}=\left\{ \begin{array}{l} 200+100*\lfloor \frac{n}{50}\rfloor ,\;n\leq 500\\ 2000+500*\lfloor \frac{n-500}{100}\rfloor ,\;500\leq n<1000\\ 5000+2500*\lfloor \frac{n-1000}{200}\rfloor ,\;n\geq 1000 \end{array}\right.$$ (15)

$$n_{t}=\left\{ \begin{array}{l} 50,\;n\leq 500\\ 20,\;500\leq n<1000\\ 10,\;n\geq 1000 \end{array}\right.$$ (16)

Table 2 lists *min* (*S*_*min*_), *avg* (*S*_*avg*_), *max* (*S*_*max*_), *S*_*td*_, and the average time cost of the 18 instances tested by QL, ABSQL, and DSRABSQL, and the corresponding values of VDWOA and DWOA.

**Table 2:** Results of proposed algorithms and algorithms in [9] for 18 instances.

Instance (OPT)	Algorithm	*min* (*S*_*min*_ (%))	*avg* (*S*_*avg*_ (%))	*max* (*S*_*max*_ (%))	*S*_*td*_	Time(s)
Oliver30(420)	QL	422(0.48)	431.6(2.76)	442(5.24)	5.6	0.73
	ABSQL	**420(0)**	428.6(2.05)	438(4.29)	4.7	0.60
	DSRABSQL	**420(0)**	**422.4(0.57)**	**426(1.43)**	**2.1**	0.61
	VDWOA	**420(0)**	429.7(2.30)	436(3.81)	6.3	4.74
	DWOA	**420(0)**	448.8(6.85)	473(12.62)	16.8	2.08
Eil51(426)	QL	468(9.86)	516.4(21.22)	572(34.27)	33.6	1.71
	ABSQL	**428(0.47)**	443.3(4.06)	458(7.51)	8.1	1.35
	DSRABSQL	**428(0.47)**	**431.4(1.27)**	**438(2.82)**	**3.4**	1.37
	VDWOA	429(0.7)	448.9(5.37)	459(7.75)	8.1	10.47
	DWOA	445(4.46)	472.9(11.01)	482(13.15)	11.1	3.73
Berlin52(7542)	QL	7646(1.38)	7890.3(4.62)	8142(7.96)	139.7	2.57
	ABSQL	7548(0.08)	7864.8(4.28)	8035(6.54)	125.2	2.66
	DSRABSQL	**7542(0)**	**7609.5(0.89)**	**7653(1.47)**	**37.0**	2.68
	VDWOA	**7542(0)**	8026.7(6.43)	8387(11.20)	237.6	10.79
	DWOA	7727(2.45)	8473.2(12.35)	8934(18.46)	316.2	3.78
St70(675)	QL	734(8.74)	762.1(12.90)	786(16.44)	16.5	2.89
	ABSQL	684(1.33)	712.4(5.54)	724(7.26)	10.6	4.96
	DSRABSQL	**675(0)**	**685.2(1.51)**	**691(2.37)**	**4.2**	4.97
	VDWOA	676(0.15)	718.9(6.50)	742(9.93)	22.0	17.68
	DWOA	712(5.48)	761.0(12.74)	809(19.85)	27.1	5.31
Eil76(538)	QL	582(8.18)	626.4(16.43)	658(22.30)	25.6	1.30
	ABSQL	**550(2.23)**	567.4(5.46)	580(7.81)	9.4	1.18
	DSRABSQL	**550(2.23)**	**556.6(3.46)**	**562(4.46)**	**4.1**	1.20
	VDWOA	554(2.97)	584.6(8.66)	626(16.36)	21.2	20.5
	DWOA	579(7.62)	609.1(13.21)	633(17.66)	17.6	5.81
Pr76(108159)	QL	118233(9.31)	125581.4(16.11)	127746(18.11)	3311.5	4.58
	ABSQL	114523(5.88)	121951.8(12.75)	125484(16.02)	3066.2	5.15
	DSRABSQL	111363(2.96)	**112788.2(4.28)**	**113696(5.12)**	**567.1**	5.18
	VDWOA	**108353(0.18)**	114176.9(5.56)	120648(11.55)	3539.5	20.52
	DWOA	111511(3.1)	117788.2(8.90)	120367(11.29)	2658.4	5.85
KroA100(21282)	QL	23491(10.38)	25377.9(19.25)	26432(24.20)	996.8	4.62
	ABSQL	22564(6.44)	23796.5(11.82)	24550(15.26)	513.5	5.00
	DSRABSQL	**21470(0.88)**	**21637.2(1.67)**	**21782(2.35)**	**110.3**	5.04
	VDWOA	22653(2.06)	22881.9(7.52)	24193(13.68)	752.6	35.24
	DWOA	22471(5.59)	24877.1(16.86)	26572(24.86)	1138.2	8.12
Eil101(629)	QL	680(8.11)	716.7(13.94)	746(18.60)	18.6	5.5
	ABSQL	658(4.61)	692.5(10.10)	724(15.10)	20.2	8.5
	DSRABSQL	653(4.61)	**660.2(4.96)**	**667(6.04)**	**4.1**	8.6
	VDWOA	**644(2.38)**	681.8(8.40)	714(13.51)	20.1	13.55
	DWOA	683(8.59)	712.3(13.24)	745(18.44)	18.1	10.41
Ch150(6528)	QL	7124(9.13)	7321.3(12.15)	7454(14.19)	155.7	4.72
	ABSQL	6828(4.60)	6996(7.17)	7167(9.79)	122.3	5.00
	DSRABSQL	**6615(1.33)**	**6645.6(1.80)**	**6698(2.60)**	**25.9**	5.04
	VDWOA	6863(5.13)	7374.5(12.97)	7641(17.05)	209.9	77.37
	DWOA	7329(12.27)	7688.5(17.78)	7905(21.09)	182.4	13.87
D198(15780)	QL	17512(10.98)	18358.2(16.34)	18864(19.54)	394.6	16.71
	ABSQL	16782(6.35)	17420.2(10.39)	17677(12.02)	287.6	14.70
	DSRABSQL	**16157(2.39)**	**16314.7(3.39)**	**16425(4.09)**	**84**	14.72
	VDWOA	16313(3.38)	16858.1(6.83)	17097(8.35)	230.1	145.24
	DWOA	16603(5.22)	17337.2(9.87)	17568(11.33)	296.7	20.56
TSP225(3916)	QL	4266(8.94)	4682.5(19.57)	4847(23.77)	169.5	24.8
	ABSQL	4025(2.78)	4345(10.96)	4461(13.92)	112.3	38.1
	DSRABSQL	**3877(-1.00)**	**3954.2(0.98)**	**4012(2.45)**	**45.6**	38.2
	VDWOA	4136(5.62)	4362.8(11.41)	4462(13.94)	96.8	195.54
	DWOA	4399(12.33)	4665.5(19.14)	4730(20.79)	97.6	25
Pr299(48191)	QL	57326(18.96)	58652.1(21.71)	60102(24.72)	745.6	44.7
	ABSQL	54794(13.70)	55612(15.40)	56527(17.30)	539.6	58.0
	DSRABSQL	**49291(2.28)**	**49864.8(3.47)**	**50542(4.88)**	**339.6**	58.1
	VDWOA	55312(14.78)	59106.9(22.65)	60405(25.34)	1368.8	383.46
	DWOA	59187(22.82)	62081.8(28.82)	63111(30.96)	1062.9	168.27
Fl417(11861)	QL	14574(22.87)	15640.9(31.87)	16310(37.51)	554.8	51.00
	ABSQL	13037(9.91)	13435.7(13.28)	13754(15.96)	243.9	72.00
	DSRABSQL	**12206(2.91)**	**12478.2(5.20)**	**12657(6.71)**	**122.3**	72.14
	VDWOA	12462(5.07)	13191.7(11.22)	13938(17.51)	430.0	2485.61
	DWOA	13886(17.07)	15908.6(34.13)	16276(37.22)	645.3	276.12
D493(35002)	QL	42215(20.61)	45356.5(29.58)	46325(32.35)	1176.4	143.2
	ABSQL	38836(10.95)	40046(14.41)	41101(17.42)	648.1	189.2
	DSRABSQL	**36408(4.02)**	**37244.5(6.41)**	**37654(7.58)**	**295.6**	189.3
	VDWOA	38206(9.15)	39533.2(12.95)	41023(17.20)	805.3	5672.84
	DWOA	47803(36.57)	48784.5(39.38)	50246(43.55)	794.7	381.19
Rat575(6773)	QL	8286(22.34)	8854.3(30.73)	9224(36.19)	296.2	411.5
	ABSQL	7609(12.34)	7754(14.48)	7940(17.23)	128.8	446.7
	DSRABSQL	**7462(10.17)**	**7536.8(11.28)**	**7602(12.24)**	**61.0**	446.8
	VDWOA	7654(13.01)	7725.1(14.06)	8048(18.82)	73.0	11100.5
	DWOA	8190(20.92)	8440.0(24.61)	9021(33.19)	229.2	873.41
Rat783(8806)	QL	11213(27.33)	12212.8(38.69)	13025(47.91)	529.5	433.5
	ABSQL	9990(13.45)	10133(15.07)	10225(16.11)	77.2	608.6
	DSRABSQL	**9556(8.52)**	**9854.1(11.90)**	**9920(12.65)**	**41.9**	608.8
	VDWOA	10370(17.76)	10596.4(20.33)	11015(25.09)	186.1	26118.5
	DWOA	12299(39.67)	12854.3(45.97)	13425(52.45)	302.0	1638.6
Fl1400(20127)	QL	26631(32.31)	27134.2(34.81)	27540(36.83)	256.4	9842
	ABSQL	22652(12.55)	22985.5(14.20)	23186(15.20)	**171.8**	10308
	DSRABSQL	**21638(7.51)**	**21968.6(9.15)**	**22265(10.62)**	196.6	10310
	VDWOA	24036(19.42)	24886.1(23.65)	25875(28.56)	451.9	173425
	DWOA	24821(23.32)	26342.9(30.88)	27036(34.33)	734.1	19211
D1655(62128)	QL	78835(26.89)	84584.5(36.15)	87016(40.06)	2624.9	12285
	ABSQL	71113(14.46)	71707.3(15.39)	72031(15.94)	312.9	13080
	DSRABSQL	**68606(10.43)**	**69132.2(11.27)**	**69352(11.63)**	**232.3**	13083
	VDWOA	74258(19.52)	75724.3(21.88)	76987(23.92)	778.4	242326
	DWOA	75542(21.59)	76990.5(23.92)	78503(26.36)	866.1	31781

From Table 2, it can be seen that *min*, *avg*, *max*, and *S*_*td*_ of DSRABSQL are mostly better than those of VDWOA for 18 instances, which indicates that the solution quality and stability of DSRABSQL are superior to those of VDWOA.

To further analyze the performance of the algorithm, the data of Table 2 are discussed in two aspects: the first is the data comparison of QL, ABSQL and DSRABSQL, and the second is the data comparison between DSRABSQL and VDWOA.

**Fig 7:**
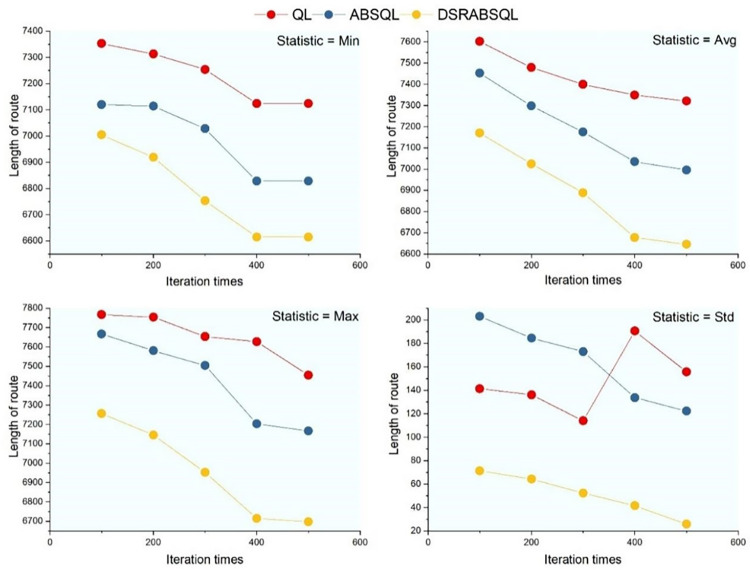
*min*, *avg*, *max* and *S*_*td*_ of ch150 obtained by three algorithms.

**Fig 8:**
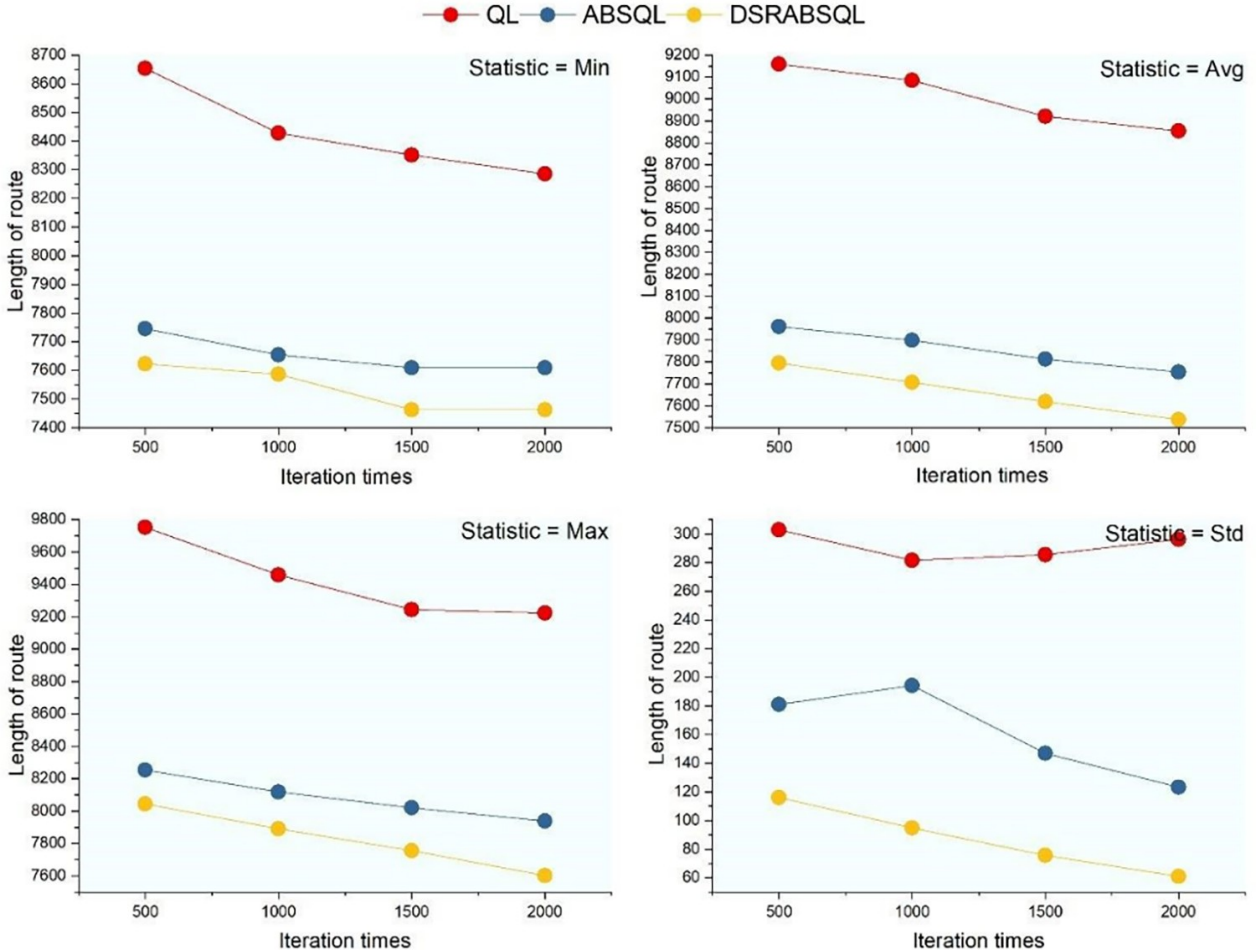
*min*, *avg*, *max* and *S*_*td*_ for rat575 obtained by three algorithms.

**Fig 9:**
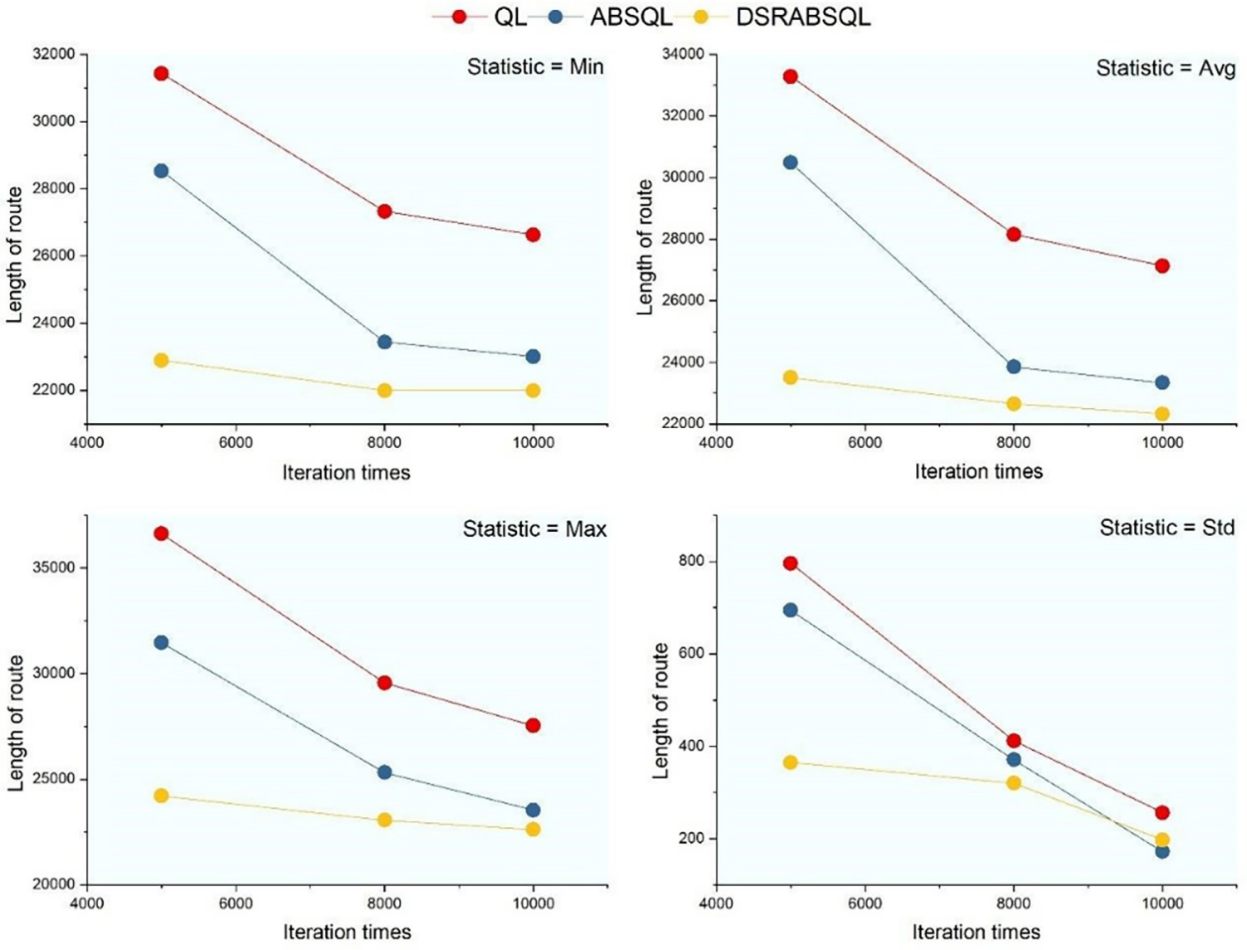
*min*, *avg*, *max* and *S*_*td*_ of fl1400 obtained by three algorithms.

For the first aspect, a small- (ch150), medium- (rat575), and large-scale (fl400) instance are selected to analyze the performance of DSRABSQL. Figs 7–9 illustrate *min*, *avg*, *max*, *S*_*td*_, and *N*_*min*_ (the number of *mins)* obtained by using QL, ABSQL, and DSRABSQL, respectively, to test ch150, rat575, and fl1400 under different numbers of iterations. Since the time cost of the algorithm will increase with scale of the instance, ch150, rat575, and fl1400 are tested 50, 20, and 10 times, respectively.

**Fig 10:**
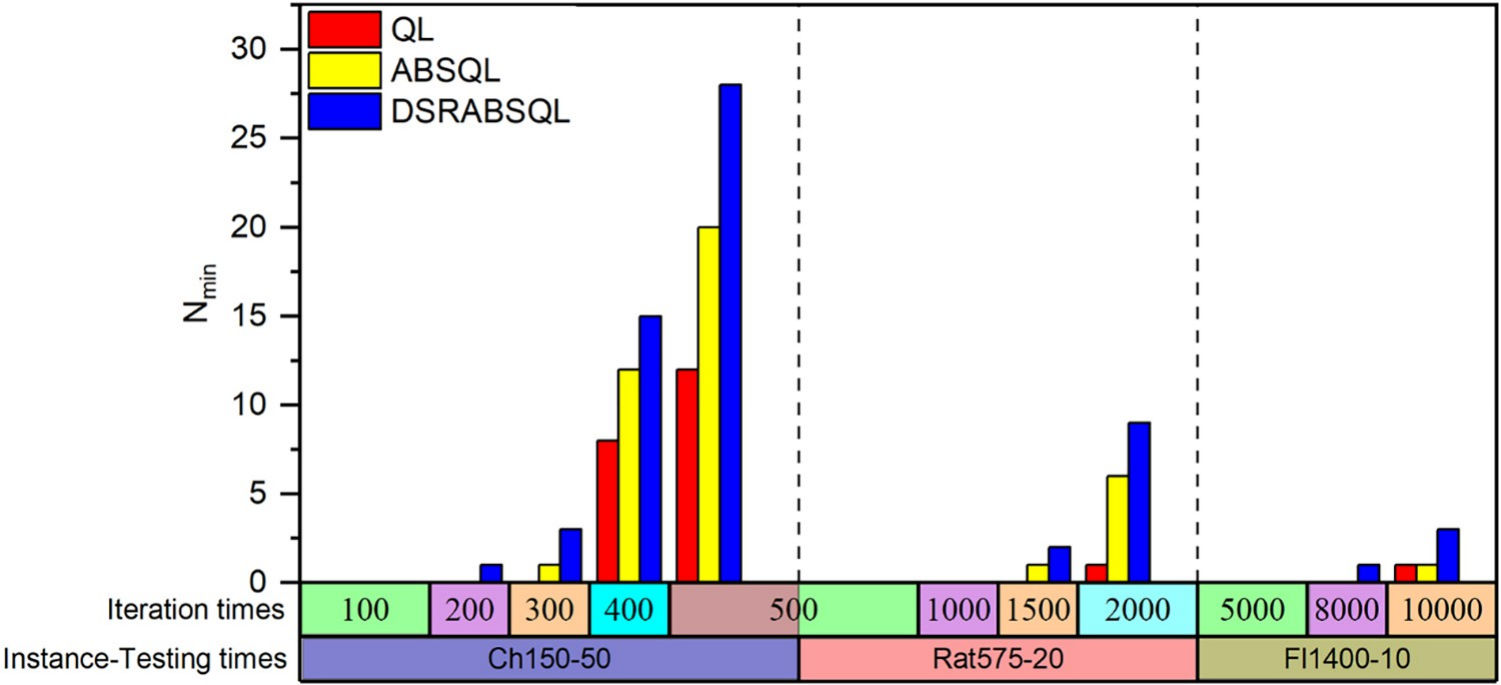
The value of *N*_*min*_ for ch150, rat575 and fl1400 obtained by three algorithms.

A chi-square test is performed on *N*_*min*_ as obtained for ch150 under 400 and 500 iterations of DSRABSQL, as shown in Fig 10, where *N*_*min*_ is the number of *mins*, and *I*_*t*_ is the number of iterations. With 1 degree of freedom, we assume Hypothesis 0: *N*_*min*_ and *I*_*t*_ are correlated.

For this purpose, the Sample Frequency Contingency Table of *N*_*min*_ and *I*_*t*_, as shown in Table 3, must be made, and a chi-square test based on the fourfold data in Table 3 is run, with the test statistic *χ*^*2*^ calculated as 6.895.

**Table 3:** Sample frequency contingency table of *N*_*min*_ and *I*_*t*_.

*I*_*t*_	*I*_*t = 400*_	*I*_*t = 500*_	Total
*N*_*min*_	15	28	43
*N*_*non_min*_	35	22	57
total	50	50	100
*χ* ^*2*^	6.895		
*χ*^*2*^(*p* = 0.99)	6.635		
*χ*^*2*^(*p* = 0.995)	7.879		

From Table 2 and Figs 7~10, it can be seen that *min*, *avg*, *max*, *S*_*td*_, and *N*_*min*_ of DSRABSQL are better than those of QL and ABSQL, which indicates that the solution quality, stability, and convergence of DSRABSQL are all significantly improved compared with QL and ABSQL throughout the iterative process. Table 3 shows that *χ*^*2*^∈(6.635, 7.879), and the probability *p* that Hypothesis 0 is true is between 0.99 and 0.995, which indicates that *N*_*min*_ and *I*_*t*_ are significantly correlated. Similarly, *N*_*min*_ and *I*_*t*_ of instances rat575 and fl1400 can also be inferred to be correlated. Therefore, *N*_*min*_ of DSRABSQL increases with *I*_*t*_ until achieving stability, i.e., the probability of DSRABSQL for getting its minimum value *min* will increase with *I*_*t*_ until stability. The above analysis shows that DSRABSQL has learning ability and can be continuously enhanced with iterations until stability.

For the second aspect, the Mann-Whitney-Wilcoxon test is used to verify the performance of DSRABSQL relative to VDWOA based on the data of Table 2. A parameter,

$$S_{c}=0.5*S_{min}+0.4*S_{avg}+0.1*S_{max},$$ (17)

is introduced to evaluate the performance of the algorithm under different scale instances. Since *S*_*min*_, *S*_*avg*_, and *S*_*max*_ have different effects on performance of the algorithm, we assign them different weights, in this case 0.5, 0.4, and 0.1, respectively. The smaller *S*_*c*_ is, the better the comprehensive performance of the algorithm is.

The values of *S*_*c*_ for 18 instances of DSRABSQL and VDWOA are calculated, with results as shown in Tables 3 and 4. To verify that DSRABSQL shows more advantages in solving medium- and large-scale instances, the 18 instances are divided into groups, *G*_*1*_ and *G*_*2*_. Group *G*_*1*_ includes 14 small-scale instances, and *G*_*2*_ includes 4 medium- and large-scale instances. In group *G*_*1*_, *S*_*c*_ of DSRABSQL is marked as Sample 1, and that of VDWOA as Sample 2; for group *G*_*2*_, *S*_*c*_ of DSRABSQL is marked as Sample 3, and that of VDWOA as Sample 4. Then, the following hypotheses are made:

Hypothesis 1(a): *S*_*c*_ of DSRABSQL in *G*_*1*_ and *G*_*2*_ is not significantly different from that of VDWOA;

Hypothesis 1(b): *S*_*c*_ of DSRABSQL in *G*_*1*_ and *G*_*2*_ differ significantly from that of VDWOA.

**Table 4:** *S*_*c*_ and rank of DSRABSQL and VDWOA algorithms in *G*_*1*_.

*G*_*1*_	Sample 1			Sample 2		
	*S*_*c*_ (%)	Rank	*R*_*1*_/*U*_*1*_	*S*_*c*_ (%)	Rank	*R*_*2*_/*U*_*2*_
Oliver30	0.371	2	132/27	1.301	6	274/169
Eil51	1.025	5		3.273	12	
Berlin52	0.503	3		3.692	15	
St70	0.841	4		3.668	14	
Eil76	2.945	9		6.585	23	
Pr76	3.704	16		3.469	13	
KroA100	1.343	7		5.406	20	
Eil101	4.498	18		5.901	22	
Ch150	1.649	8		9.458	26	
D198	2.96	10		5.257	19	
TSP225	0.137	1		8.768	24	
Pr299	3.016	11		18.984	28	
Fl417	4.206	17		8.774	25	
D493	5.332	21		11.475	27	

Then, *S*_*c*_ values of Samples 1 and 2 are sorted in ascending order. *R*_*1*_ and *R*_*2*_ are the respective rank sums of Samples 1 and 2. The Mann-Whitney-Wilcoxon test is performed on *R*_*1*_ and *R*_*2*_ to obtain statistics *U*_*1*_ and *U*_*2*_, and the results are shown in Table 4. Similarly, *R*_*3*_ and *R*_*4*_ are the respective rank sums of Samples 3 and 4, respectively, and *U*_*3*_ and *U*_*4*_ are obtained by performing the same process; the results are shown in Table 5.

**Table 5:** *S*_*c*_ and rank of DSRABSQL and VDWOA algorithm in *G*_*2*_.

*G*_*2*_	Sample 3			Sample 4		
	*S*_*c*_ (%)	Rank	*R*_*3*_/*U*_*3*_	*S*_*c*_ (%)	Rank	*R*_*4*_/*U*_*4*_
Rat575	10.436	3	10/0	14.011	5	26/25
Rat783	10.285	2		19.521	6	
Fl1400	10.238	1		22.026	8	
D1655	11.011	4		20.904	7	

For *U*_*1*_ and *U*_*3*_ of Tables 4 and 5, it can be found that *U*_*1*_ = 27<47 = *U*_*α = 0*.*02*_<*U*_*α = 0*.*025*_ by checking the Mann-Whitney Table. *U*_*1*_ is lower than *U*_*α*_ after Bonferroni correction, *U*_*3*_ = 0 = *U*_*α = 0*.*05*_, so the probability *p* that Hypothesis 1(b) is true is 0.95. This shows that DSRABSQL algorithm has significant advantages in all scale instances compared with VDWOA.

**Fig 11:**
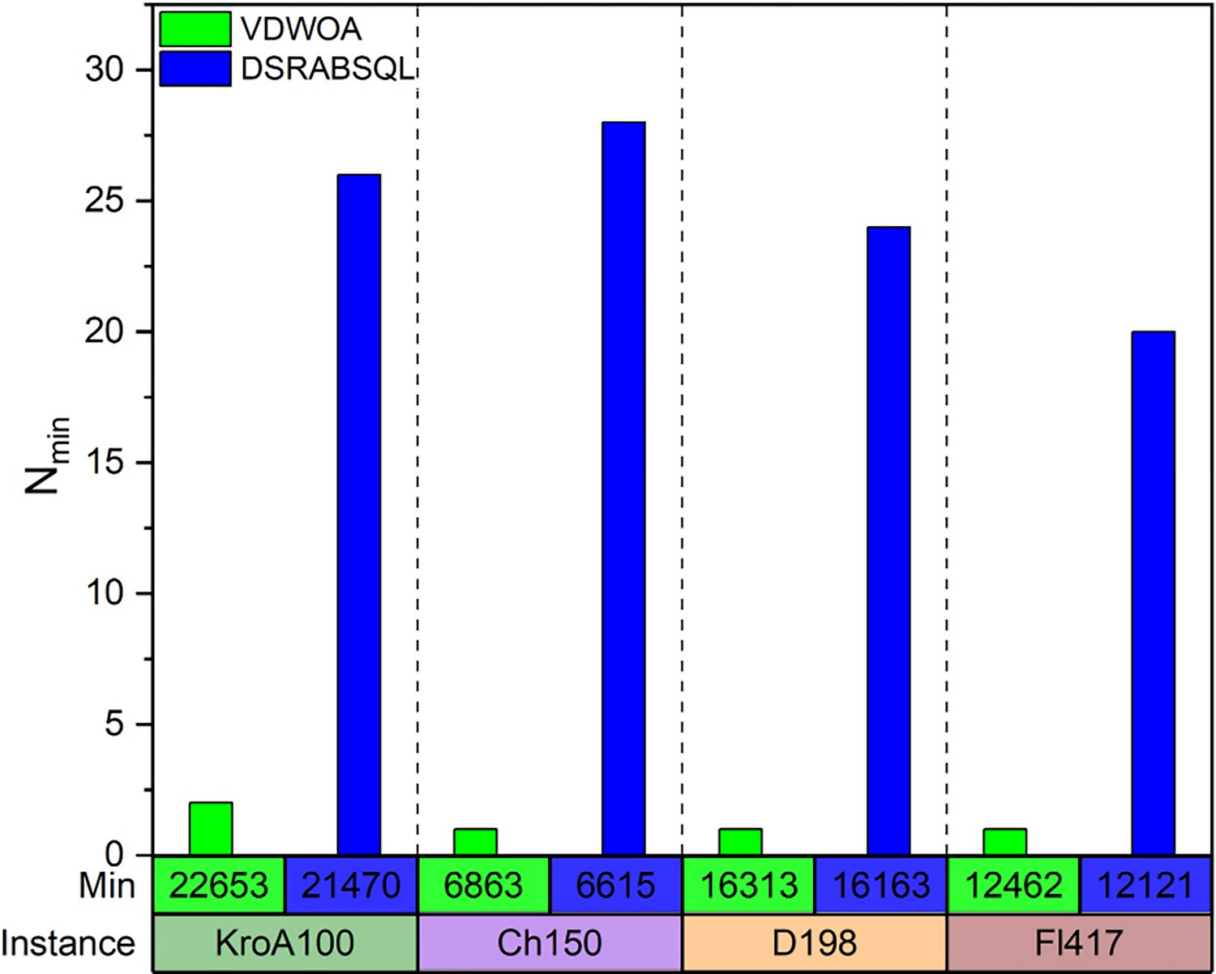
The *N*_*min*_ of DSRABSQL and VDWOA algorithms obtained by testing kroa100, ch150, d198, fl417 50 times, respectively.

Fig 11 also shows *N*_*min*_ as obtained by testing instances kroa100, ch150, d198, and fl417 for 50 times by DSRABSQL and VDWOA under the condition of convergence. The results show that *min* of DSRABSQL is better than that of VDWOA, and when the algorithms converge, DSRABSQL has a much higher probability of obtaining *min*.

To further verify the significance difference of DSRABSQL and VDWOA for groups G1 and G2, Vargha and Delaney’s *Â* was used to compute its effect size, and the following results are obtained: *Â*_*21*_ = 0.86>0.71 and *Â*_*43*_ = 1>0.71. This means that probability of the DSRABSQL algorithm is better than VDWOA for instances of all scales is about 0.86–1. Combined with the conclusions obtained from Fig 10 and Table 2, it can be further concluded that DSRABSQL has better learning ability than the population- and random search-based VDWOA.

The above analysis shows that performance of DSRABSQL is a significant improvement compared with QL, ABSQL, and VDWOA, because DSRABSQL undergoes two stages of improvement based on the QL algorithm. The first stage is from QL to ABSQL, with the proposed *stgy _1*–*stgy_4* strategies, which are all improvement methods within the QL framework. The second stage is from ABSQL to DSRABSQL, with the proposed *stgy_5* strategy, which is an improvement method outside the QL framework. We compare the improvement effects of these strategies in what follows.

**Fig 12:**
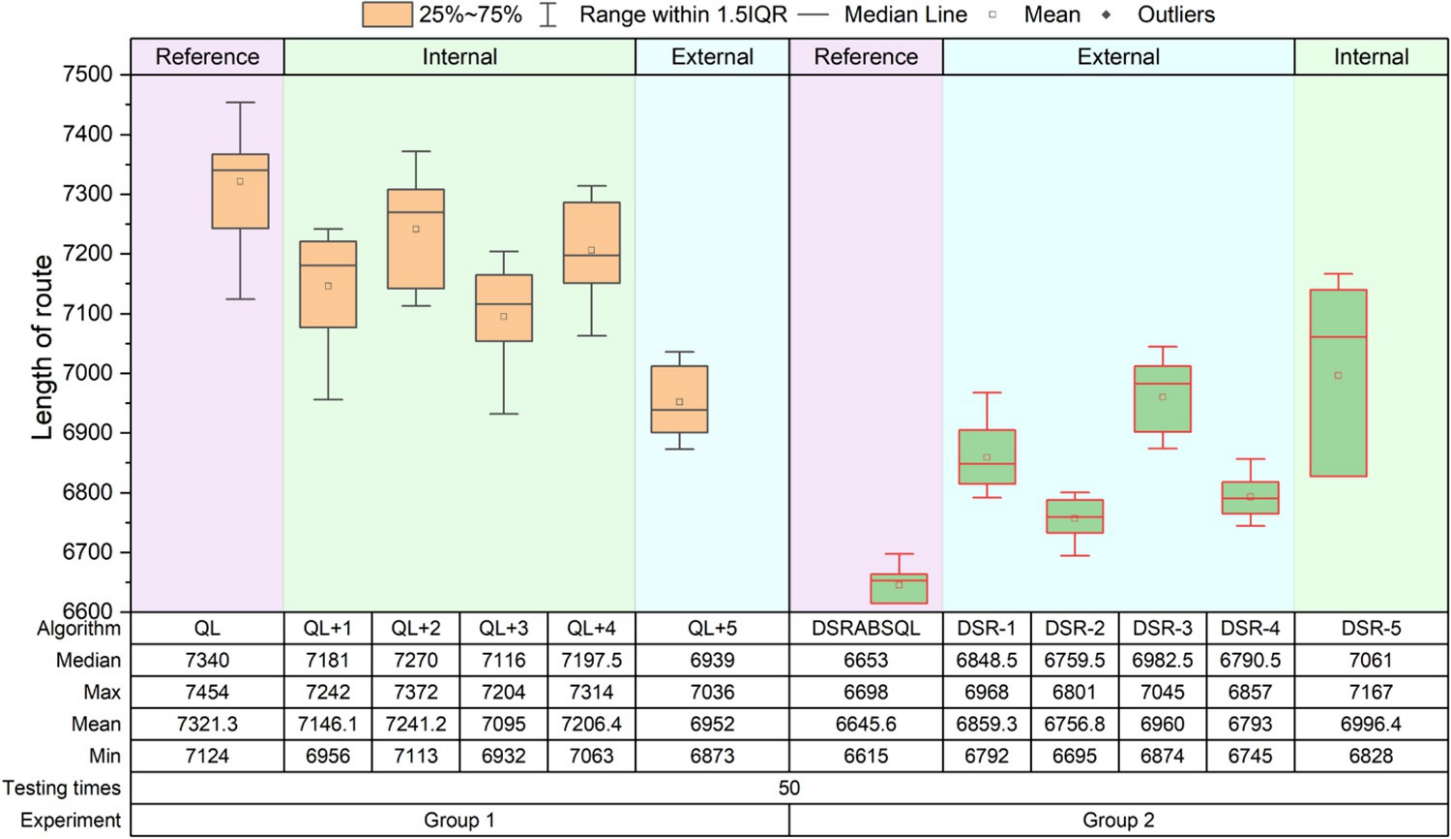
Boxplots obtained by testing ch150 for 50 times by each algorithm.

**Fig 13:**
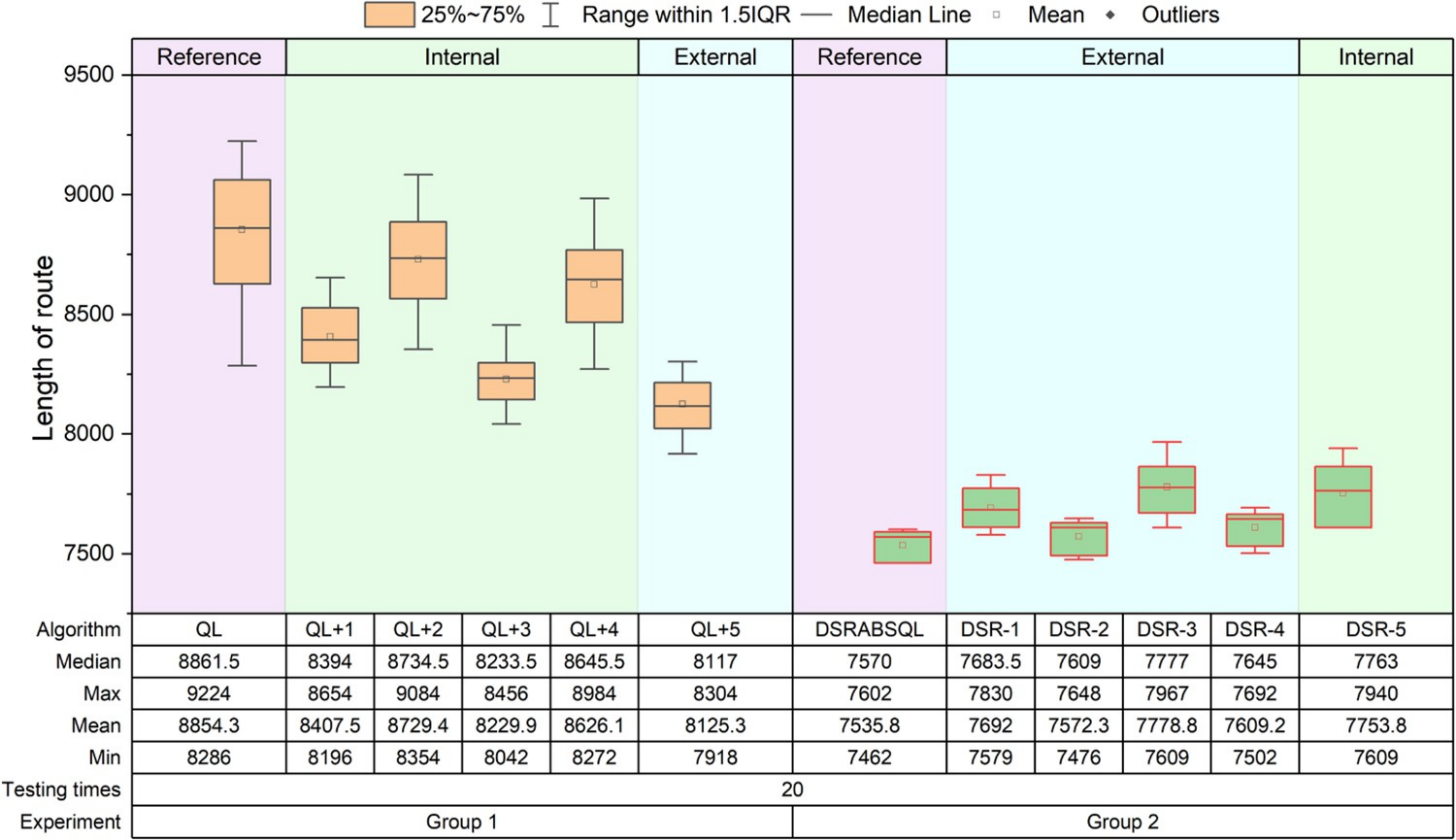
Boxplots obtained by testing rat575 for 20 times by each algorithm.

**Fig 14:**
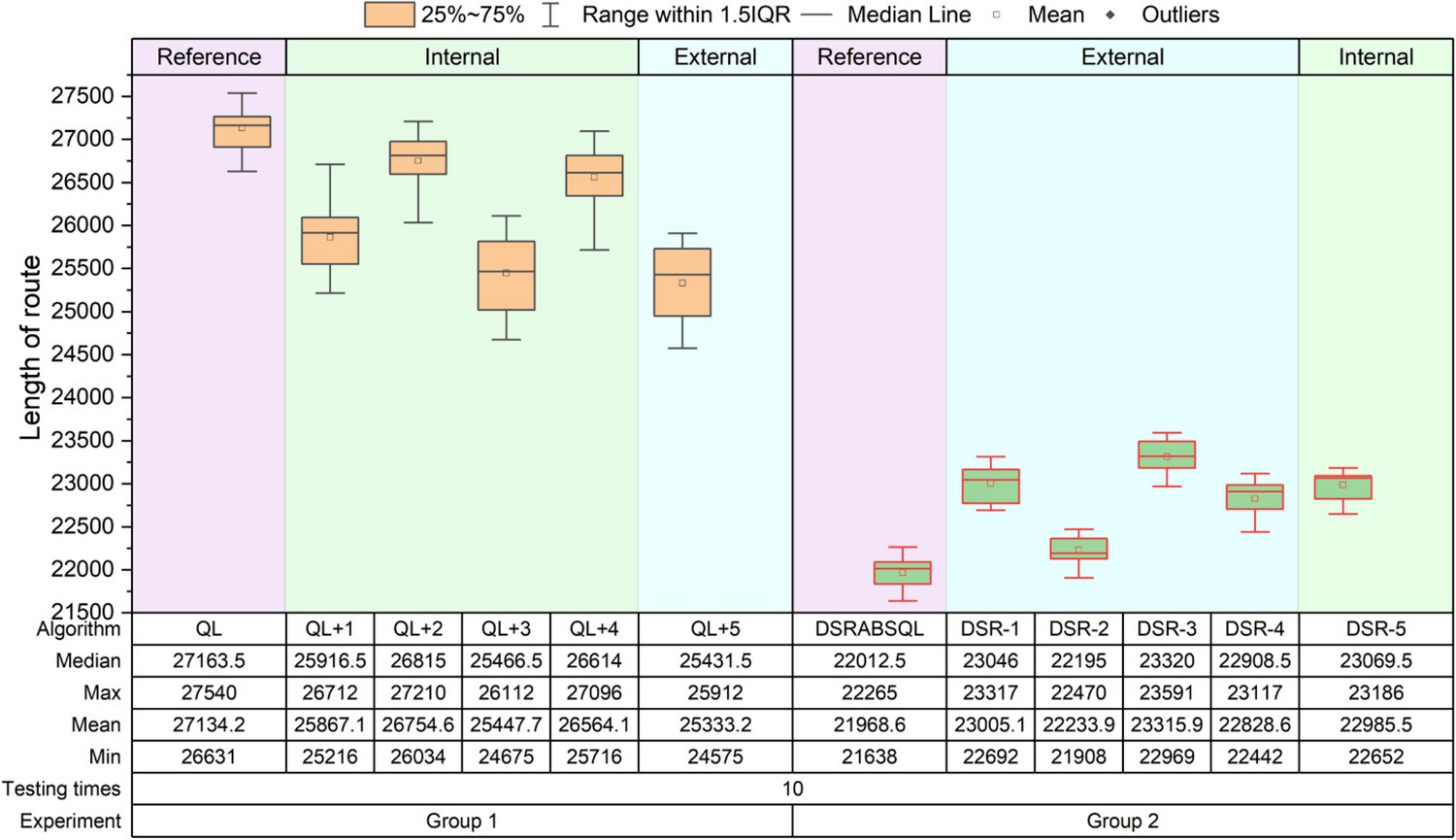
Boxplots obtained by testing fl1400 for 10 times by each algorithm.

1. Two groups of comparative experiments are designed. Group 1 includes QL and five comparison algorithms, which are obtained by combining one of the five strategies above with QL, and denoted as *QL+1*,…, *QL+4*, *QL +5*. *QL+1–QL+4* are improvements within the QL framework, and *QL+5* is an improvement outside the QL framework. Group 2 includes DSRABSQL and five comparison algorithms, which are obtained by removing one of the five strategies above from DSRABSQL, and denoted as *DSR-1*,…, *DSR-4*, *DSR-5*. *DSR-1–DSR-4* are improvements outside the QL framework, and *DSR-5* is an improvement within the QL framework. The relationships between the algorithms and the five strategies are shown in Table 6.
2. For every algorithm of each group, instances ch150, rat575, and fl1400 are tested 50, 20, and 10 times, respectively. The experiment results of ch150, rat575, and fl1400 are illustrated by the boxplots of Figs 12–14, respectively.
3. For each comparison algorithm, according to the position distribution of the boxes shown in these figures, the effects of these strategies on improvement of the algorithms are judged. The values of *min*, *avg* and *max* in the boxplot are converted to *S*_*c*_ for processing, and the results are shown in Table 7.

**Table 6:** Table of relationships between algorithms and five strategies for two experimental groups.

Algorithm	Internal Strategy				External Strategy
	*Stgy_1*	*Stgy_2*	*Stgy_3*	*Stgy_4*	*Stgy_5*
QL+1	√	—	—	—	—
QL+2	—	√		—	—
QL+3	—	—	√	—	—
QL+4	—	—	—	√	—
QL+5	—	—	—	—	√
DSR-1	—	√	√	√	√
DSR-2	√	—	√	√	√
DSR -3	√	√	—	√	√
DSR -4	√	√	√	—	√
DSR -5	√	√	√	√	—

**Table 7:** *S*_*c*_ of ch150, rat575 and fl1400 for algorithms of each group.

Instance	Group 1		Group 2	
	Algorithm	*S*_*c*_ (%)	Algorithm	*S*_*c*_ (%)
Ch150	QL+1	8.16	DSR-1	4.73
	QL+2	10.15	DSR-2	3.10
	QL+3	7.61	DSR -3	6.09
	QL+4	9.46	DSR -4	3.79
	QL+5	6.02	DSR -5	6.15
Rat575	QL+1	22.93	DSR-1	12.94
	QL+2	26.64	DSR-2	11.20
	QL+3	20.46	DSR -3	13.87
	QL+4	25.27	DSR -4	11.68
	QL+5	18.70	DSR -5	13.69
Fl1400	QL+1	27.32	DSR-1	13.68
	QL+2	31.37	DSR-2	9.78
	QL+3	24.85	DSR -3	15.12
	QL+4	30.14	DSR -4	12.60
	QL+5	24.27	DSR -5	13.48

According to Figs 12~14, two kinds of comparisons are performed, as follows:

(1) Horizontal comparison

For instance, for ch150 in experimental Group 1, the improvement degrees of the *QL+2*, *QL+4*, *QL+1*, *QL+3*, and *QL+5* algorithms increase sequentially compared with QL. In experimental Group 2, the performance of *DSR-2*, *DSR-4*, *DSR-1*, *DSR-3*, and *DSR-5* deteriorates gradually compared with DSRABSQL.

For rat575 and fl1400 in experimental Group 1, the improvement degree of *QL+2*, *QL+4*, *QL+1*, *QL+3*, and *QL+5* increases sequentially compared with QL. In experimental Group 2, the performance of *DSR-2*, *DSR-4*, *DSR-1*, *DSR-5*, and *DSR-3* deteriorates gradually compared with DSRABSQL.

(2) Vertical comparison

In experimental Group 1, for ch150, rat575, and fl1400, the improvement degree of *QL+2*, *QL+4*, *QL+1*, *QL+3*, and *QL+5* increases sequentially compared with QL, and with expansion of the instance scale, the solution quality of *QL+3* is constantly approaching that of *QL+5*.

In experimental Group 2, for ch150, the performance of *DSR-2*, *DSR-4*, *DSR-1*, *DSR-3*, and *DSR-5* deteriorates gradually compared with DSRABSQL. For rat575 and fl1400, the performance of *DSR-2*, *DSR-4*, *DSR-1*, *DSR-5*, and *DSR-3* deteriorates gradually compared with DSRABSQL. It can be also found that, with the increase of the instance scale, the gap between *DSR-3* and the other four algorithms increases gradually, but the solution quality of *DSR-1* and *DSR-4* constantly approaches that of *DSR-5*.

The above analysis shows that for small-scale instances, *stgy_5* makes the greatest contribution to QL and DSRABSQL, indicating its key role in early and later improvement stages of the algorithm. For medium- and large-scale instances, *stgy_5* contributes most to the QL algorithm, and *stgy_3* contributes most to DSRABSQL, which indicates that *stgy_5* plays a key role in the early improvement stage of the algorithm, and that *stgy_3* plays a key role in the later improvement stage. In conclusion, *stgy_5* has the greatest potential for small-scale instances, and *stgy_3* for medium- and large-scale instances. *stgy_1* and *stgy_4* also have certain potential for large-scale instances, while *stgy_2* has no obvious influence for any scale of instances.

In addition, from the difference of *S*_*c*_ of each algorithm in Table 7, it can be found that there are collaborations between strategies. To describe this phenomenon in detail, the following data are calculated based on Table 7. Since *stgy_5* is the only one of the five strategies outside the QL framework, and it has the best improvement without the collaboration of other strategies, we use this as the reference strategy. The difference of *S*_*c*_ between *QL+5* and *QL*+*k* (*k*∈{1,2,3,4}) is denoted as *d*_*5-k*_, which is calculated as

$$d_{5-k}=S_{c}^{QL+k}-S_{c}^{QL+5},$$ (18)

and the difference of *S*_*c*_ between *DSR-5* and *DSR-k* is denoted as *d´*_*5-k*_, which is calculated as

$${{d´}_{5-k}=S}_{c}^{DSR-5}-S_{c}^{DSR-k},$$ (19)

where *S*_*c*_^*QL*+*k*^, *S*_*c*_^*QL*+*5*^, *S*_*c*_^*DSR-k*^, and *S*_*c*_^*DSR-5*^ represent the *S*_*c*_ value of the corresponding instances of *QL*+*k*, *QL*+*5*, *DSR-k*, and *DSR-5*, respectively. For algorithm *QL*+*k*, no collaboration exists between *stgy_1*~ *stgy_5*, but it does for *DSR-k*. These algorithms are divided into four classes according to their adopted strategies and the relevant data are listed in Table 8.

**Table 8:** The difference of *S*_*c*_ between *stgy_5* and *stgy_1*~ *stgy_4* under collaboration or not.

Class Number	Algorithm	Adopted Strategies	Difference of *S*_*c*_		
			Ch150	Rat575	Fl1400
I	QL+1	*stgy_1*	2.14	4.23	3.05
	QL+5	*stgy_5*			
	DSR -5	*stgy_2*, *stgy_3*, *stgy_4 and stgy_1*	1.42	0.75	-0.20
	DSR -1	*stgy_2*, *stgy_3*, *stgy_4 and stgy_5*			
II	QL+2	*stgy_2*	4.13	7.94	7.10
	QL+5	*stgy_5*			
	DSR -5	*stgy_1*, *stgy_3*, *stgy_4 and stgy_2*	4.03	2.49	3.70
	DSR -2	*stgy_1*, *stgy_3*, *stgy_4 and stgy_5*			
III	QL+3	*stgy_3*	1.59	1.76	0.58
	QL+5	*stgy_5*			
	DSR -5	*stgy_1*, *stgy_2*, *stgy_4 and stgy_3*	0.06	-0.18	-1.64
	DSR -3	*stgy_1*, *stgy_2*, *stgy_4 and stgy_5*			
IV	QL+4	*stgy_4*	3.44	6.57	5.87
	QL+5	*stgy_5*			
	DSR -5	*stgy_1*, *stgy_2*, *stgy_3 and stgy_4*	2.36	2.01	0.88
	DSR -4	*stgy_1*, *stgy_2*, *stgy_3 and stgy_5*			

From Table 8, the following results can be found. For ch150 of class I, *S*_*c*_ of QL combined with *stgy_1* (*QL+1*) is 2.14% larger than that of QL combined with *stgy_5* (*QL+5*); however, the *S*_*c*_ of QL combined with *stgy_1*, *stgy_2*, *stgy_3* and *stgy_4* (*DSR-5*) is 1.42% larger than that of QL combined with *stgy_2*, *stgy_3*, *stgy_4* and *stgy_5* (*DSR-1*), which indicates that with the collaboration of *stgy_2*, *stgy_3* and *stgy_4*, the difference of the improvement effect between *stgy_1* and *stgy_5* is decreased gradually. The same is found for classes II–IV. For rat575 and fl1400, similar results can also be deduced.

In other words, the improvement effect of *stgy_1*–*stgy_4* is all worse than stgy_5 when no collaboration exists. However, under collaboration, the difference of the improvement effect between *stgy_1*–*stgy_4* and *stgy_5* gradually decreases. In addition, the improvement effect of *stgy_1* and *stgy_3* is sometimes better than that of *stgy_5*, which indicates a better collaboration effect from *stgy_1*–*stgy_4*, but *stgy_5* is relatively independent.

In class I, for ch150, the *S*_*c*_ of *DSR-5* is 1.42% larger than that of *DSR-1*; for rat575, the *S*_*c*_ of *DSR-5* is 0.75% larger than that of *DSR-1*; and for fl1400, the *S*_*c*_ of DSR-5 is 0.2% less than that of *DSR-1*. This all indicates that with the expansion of the instance scale and with the collaboration of *stgy_2*, *stgy_3*, and *stgy_4*, the difference of the improvement effect between *stgy_1* and *stgy_5* decreases until it overtakes that of *stgy_5*. The same is found for classes II–IV. Therefore, the collaboration effect between *stgy_1*–*stgy_4* is approaching being better than that of *stgy_1*–*stgy_5’s* increase with instance scale.

The above analysis shows that DSRABSQL has certain advantages in solving the TSP. To further verify the effectiveness of the algorithm, from categories 1 and 2 described in section 1, we select S2V-DQN and the 2-opt heuristic algorithm combined with deep reinforcement learning (2-opt-DRL for short) from each category to compare with DSRABSQL. Table 9 lists the *min* and average of *S*_*min*_ obtained by DSRABSQL and these two algorithms for 25 instances.

**Table 9:** The *min* obtained by DSRABSQL, S2V-DQN and 2-opt-DRL algorithms for 25 instances.

Instance	*Opt*	DSRABSQL	S2V-DQN	2-opt-DRL
Eil51	426	**428**	439	427
Berlin52	7542	**7542**	**7542**	7974
St70	675	**675**	696	680
Eil76	538	**550**	564	552
Pr76	108159	111363	**108446**	111085
KroA100	21282	**21470**	21897	23751
KroC100	20749	**20937**	21074	22672
KroE100	22068	**22549**	22913	23253
Eil101	629	653	659	**635**
Lin105	14379	**14416**	15023	16153
Pr107	44303	**44303**	45113	54378
Pr124	59030	59731	61623	**59513**
Bier127	118282	**120304**	121576	121122
Ch130	6110	6185	6270	**6175**
Pr136	96772	100789	99474	**98453**
Pr144	58537	**58761**	59436	61207
Ch150	6528	6615	6985	**6597**
Pr152	73682	**75274**	75283	75301
U159	42080	**42254**	45433	42716
Rat195	2323	**2374**	2581	2955
KroA200	29368	**30241**	30965	32522
Tsp225	3916	**3877**	4154	4354
Pr226	80369	**80725**	81873	91560
A280	2579	**2623**	2867	2898
Pr299	48191	**49291**	51895	59422
**Average of *S***_***min***_ **(%)**	0.00	**1.39**	4.21	7.53

For these 25 instances, compared with S2V-DQN, the results of 23 instances obtained by DSRABSQL are better, and compared with 2-opt-DRL, those of 19 instances are better. In general, the average of *S*_*min*_ for DSRABSQL is 0.33 times that of S2V-DQN and 0.18 times that of 2-opt-DRL, which shows that the solution results of DSRABSQL still have a clear advantage compared with those of RL.

## 5. Conclusion and future work

We proposed a dynamic sub-route-based self-adaptive beam search Q-learning algorithm for medium- to large-scale TSPs. On the basis of the QL algorithm, a weighting function-based reward matrix, a power function-based initial Q-table, a self-adaptive *ε-beam* search strategy, a new Q-value update formula, and a dynamic sub-route optimization strategy were introduced. The first four are improvement strategies within the QL framework, and the last is outside of it.

Considering the comparative algorithms, the DSRABSQL algorithm not only has certain advantages in solution time, results, and solution stability, but strong local optimization and good learning abilities. However, there are certain deficiencies compared with the current best heuristic algorithms, such as LKH and DEACO. Therefore, it is necessary to further improve DSRABSQL.

For the five proposed improvement strategies for DSRABSQL, the experimental results show that they can accelerate the convergence speed of the algorithm, enhance its search and local optimization ability, and balance exploration and exploitation. In addition, there are strong collaboration effects among the four strategies within the QL framework, which will become stronger with expansion of the instance scale. Although the dynamic sub-route optimization strategy has the most obvious improvement effect on small-scale instances, its relative independence will lead to a fixed improvement effect, which will be limited as the problem size increases. The self-adaptive *ε-beam* search strategy cannot only make the greatest contributions to medium- and large-scale problems; it has strong collaboration effects with other strategies within the QL framework, and its advantage can be enhanced with increase of the instance size. Therefore, further improvement to the self-adaptive *ɛ-beam* search strategy will be studied.

DSRABSQL still faces challenges for solving larger-scale problems. Moreover, the many artificial parameters increase the complexity of parameter adjustment and may result in poor solutions for certain types of problems.

In future work, we hope to improve the self-adaptive *ε-beam* search strategy, enhance the collaboration effects between strategies, and adjust the parameters of each strategy to find a robust QL framework to improve the solution quality of large-scale problems and obtain a model with a better solution capability.
